# Barriers and facilitators to implementing evidence-based guidelines in long-term care: a qualitative evidence synthesis

**DOI:** 10.1186/s13012-021-01140-0

**Published:** 2021-07-09

**Authors:** Caitlin McArthur, Yuxin Bai, Patricia Hewston, Lora Giangregorio, Sharon Straus, Alexandra Papaioannou

**Affiliations:** 1 Dalhousie University, 5869 University Avenue, Halifax, Nova Scotia B3H 4R2 Canada; 2 GERAS Centre for Aging Research, 88 Maplewood Avenue, Hamilton, Ontario L8M 1W9 Canada; 3 Master University, 1280 Main Street West, Hamilton, Ontario L8S 4L8 Canada; 4 University of Waterloo, 200 University Avenue West, Waterloo, Ontario N2L 3G1 Canada; 5 Schlegel-UW Research Institute for Aging, 150 Laurelwood Drive, Waterloo, Ontario N2J 0E2 Canada; 6 University of Toronto, 27 King’s College Circle, Toronto, Ontario M5S 1A4 Canada

**Keywords:** Long-term care, Barriers, Facilitators, Evidence-based, Guidelines, Knowledge translation

## Abstract

**Background:**

The long-term care setting poses unique challenges and opportunities for effective knowledge translation. The objectives of this review are to (1) synthesize barriers and facilitators to implementing evidence-based guidelines in long-term care, as defined as a home where residents require 24-h nursing care, and 50% of the population is over the age of 65 years; and (2) map barriers and facilitators to the Behaviour Change Wheel framework to inform theory-guided knowledge translation strategies.

**Methods:**

Following the guidance of the Cochrane Qualitative and Implementation Methods Group Guidance Series and the ENTREQ reporting guidelines, we systematically reviewed the reported experiences of long-term care staff on implementing evidence-based guidelines into practice. MEDLINE Pubmed, EMBASE Ovid, and CINAHL were searched from the earliest date available until May 2021. Two independent reviewers selected primary studies for inclusion if they were conducted in long-term care and reported the perspective or experiences of long-term care staff with implementing an evidence-based practice guideline about health conditions. Appraisal of the included studies was conducted using the Critical Appraisal Skills Programme Checklist and confidence in the findings with the GRADE-CERQual approach.

**Findings:**

After screening 2680 abstracts, we retrieved 115 full-text articles; 33 of these articles met the inclusion criteria. Barriers included time constraints and inadequate staffing, cost and lack of resources, and lack of teamwork and organizational support. Facilitators included leadership and champions, well-designed strategies, protocols, and resources, and adequate services, resources, and time. The most frequent Behaviour Change Wheel components were physical and social opportunity and psychological capability. We concluded moderate or high confidence in all but one of our review findings.

**Conclusions:**

Future knowledge translation strategies to implement guidelines in long-term care should target physical and social opportunity and psychological capability, and include interventions such as environmental restructuring, training, and education.

**Supplementary Information:**

The online version contains supplementary material available at 10.1186/s13012-021-01140-0.

Contributions to the literature

- Evidence-based guidelines enhance the provision of care. However, trial-and-error-based approaches to implementation are costly and ineffective.
- This review summarizes knowledge on contextual factors in the long-term care setting that influence implementation of evidence-based guidelines to facilitate more effective and sustainable uptake in practice.
- By placing the findings of our qualitative evidence synthesis within the context of a behaviour change framework, our work provides theory-guided strategies to inform future translation of evidence into practice in long-term care homes.

## Background

### Description of the topic

Evidence-based guidelines summarize the best available research on health care practices to enhance the provision of consistent and appropriate care [1]. However, bringing evidence into clinical practice is an ongoing challenge. Systematic reviews on guideline adherence and utilization found that a large percentage of available guidelines do not have sustained implementation where appropriate [2, 3]. For example, an organization may implement a new guideline into practice, but the behaviours associated with it do not continue after initial introduction. In contrast, if new evidence emerges, suggesting current practices are not effective, they must be de-adopted. Guideline implementation into routine healthcare can be unpredictable, and trial-and-error approaches have been costly and ineffective, producing variable results of guideline dissemination and implementation [4, 5]. Consequently, there has been increasing interest in employing theories, models, and frameworks to direct guideline implementation. Knowledge translation focuses on developing ways to efficiently and effectively translate evidence-based knowledge into clinical care. Theory-based guideline implementation is desirable as it ensures the implementation plan and processes consider complex factors that influence success of guideline uptake prior to implementation. In this way, implementers navigate around potential pitfalls to successful implementation by conscientiously accounting for previously identified factors which could hinder their success.

Many existing knowledge translation frameworks guide researchers to consider complex factors that influence the success of guideline uptake prior to the implementation process [6–8]. The Behaviour Change Wheel is one framework that prompts users to select knowledge translation interventions based on physical, social, psychological, and environmental factors that influence the capability, opportunity, and motivation needed for behaviour change (COM-B) [7]. Central to the Behaviour Change Wheel, the COM-B system incorporates *C*apability, *O*pportunity, and *M*otivation as sources of *B*ehaviour. Users can determine what needs to change for the desired behaviour (e.g., guideline implementation) to occur by identifying barriers and facilitators and mapping them onto the COM-B system. The Behaviour Change Wheel then guides users to select potential knowledge translation interventions based on their COM-B analysis [7]. Therefore, by studying barriers and facilitators in a context-specific environment, interventions can be designed in a theory-informed manner which increases the potential for sustainable practice change.

### Why is it important to do this review?

The need to effectively translate evidence-based guidelines into practice is especially pressing for older adults [9] as the proportion of the population aged 65 years and over is growing exponentially [10]. Older adults with complex needs and comorbidities often live in long-term care (LTC) homes, which are living spaces for adults who have significant health challenges to receive access to 24-h nursing and personal care [11]. Guidelines have been developed for various health conditions in LTC homes ranging from diabetes to pressure ulcer prevention [12]. However, most knowledge translation studies on guideline implementation for older adults do not include LTC homes [13]. Knowledge translation strategies from other settings are poorly transferable to LTC because of the skill mix of the staff, environment, complexity of the residents’ conditions, and availability of resources [14]. Knowledge translation strategies must be specifically designed for LTC given the unique context of health care provision in this setting. While barriers and facilitators to guideline implementation have been systematically reviewed in other healthcare settings [13, 15], no such analyses have been conducted for the LTC sector.

### How this review might inform what is already known in this area

The findings of our study will synthesize barriers and facilitators to evidence-based guideline implementation across health conditions in LTC and mapped onto the COM-B components. Our identified barriers and facilitators and suggested knowledge translation strategies based on the COM-B mapping can be used to design theory-guided knowledge translation interventions in LTC. This will save time, effort, and resources in identifying barriers and facilitators so that planners can design interventions more quickly and efficiently. Further, our review will identify gaps in research related to evidence-based guideline implementation in LTC and make suggestions for future work.

### Objectives

The objectives of this qualitative evidence synthesis are to (1) synthesize barriers and facilitators that LTC staff experience during the implementation of evidence-based guidelines and (2) map the identified barriers and facilitators to the central component of the Behaviour Change Wheel framework to inform future theory-guided knowledge translation intervention development in the LTC setting. Our research question is “What are the barriers and facilitators to implementing evidence-based health care guidelines in LTC homes from the perspectives of staff (e.g., nurses, health care aides, physicians)?” The phenomena of interest is implementation of health care guidelines into practice and the factors that hinder or facilitate implementation. The context is LTC homes who provide 24-h nursing care for mostly frail, medically complex older adults across the world in the 21st century.

## Methods

We conducted a qualitative evidence synthesis following the guidance of the Cochrane Qualitative and Implementation Methods Group Guidance Series [16] and the ENTREQ reporting guidelines (Checklist can be found in Additional file 1) [17].

### Criteria for considering studies for this review

#### Types of studies

We included primary studies that use qualitative study designs such as ethnography, phenomenology, case studies, grounded theory studies, and qualitative process evaluations. We included studies that use both qualitative methods for data collection (e.g., focus group discussions, individual interviews, observation, diaries, document analysis, open-ended survey questions) and qualitative methods for data analysis (e.g., thematic analysis, framework analysis, grounded theory). We included studies that collect data using qualitative methods but do not analyse these data using qualitative analysis methods (e.g., open-ended survey questions where the response data are analysed using descriptive statistics only) as long as the results or findings identify barriers and facilitators as described below. We only included published studies written in English. We did not exclude studies based on our assessment of methodological limitations. We used this information about methodological limitations to assess our confidence in the review findings.

#### Target behaviour

The target behaviour was implementing evidence-based guidelines into practice (e.g., pressure injury management, pain, fractures, deprescribing). Barriers were defined as any factors that obstruct the capacity for LTC staff and homes to implement guidelines, while facilitators were any factors that enable implementation.

#### Participants

The group required to perform the target behaviour was LTC staff which included personal support workers, clinicians (e.g., nurses, physicians, pharmacists, dieticians, physiotherapists), and home administration (e.g., directors of care).

#### Setting

Studies were included if they were conducted in LTC, defined as a home where residents require 24-h nursing care, and 50% of the population is over the age of 65 years.

#### Search methods for identification of studies

Relevant articles were identified through a pre-planned literature search in MEDLINE Pubmed (1946 to present), EMBASE Ovid (1974 to present), and CINAHL (1981 to present) in July 2019 and updated in 2021. The key concepts used in the searches were “long-term care”, “guidelines”, “implementation”, “barriers”, and “facilitators”. The key concepts were combined with the Boolean operator AND, and the search words within each concept were combined with OR. The full search strategy can be found in Additional file 2.

#### Selection of studies

All titles and abstracts were screened by two team members (CM and YB) using a pilot-tested form and were included if they met our inclusion criteria as described above. We excluded articles that were not written in English, reported on implementation of guidelines that were not evidence-based (i.e., the article did not demonstrate that the guideline was developed through systematic review of literature), clinical commentaries, editorials, legal cases, letters, newspaper articles, abstracts, or unpublished literature. After title and abstract screening, the full texts of relevant articles were screened independently by the same two reviewers using a pilot-tested form. Disagreements were arbitrated by a third party.

### Data extraction

Two team members (CM and YB) independently extracted and charted the following data in duplicate using a pilot-tested data extraction form: study description (title, author, country, province/state/region, design, objectives, data collection methods, data analysis methods, name of guidelines examined, health topic of guideline examined, behaviour change framework, model, or theory used), individual participant description (profession(s), number, mean age, sex, sampling technique, response rate), LTC home description (number, size, ownership, rurality), and results/findings (identified barriers and facilitators). Data for the study results were extracted verbatim from the text under the heading “results” or “findings” where authors identified barriers and facilitators (or a synonym, e.g., challenges or supports for change) to implementation of the guidelines examined.

### Assessing the methodological limitations of included studies

The validity, robustness, and applicability of each included study was appraised by two team members (CM and PH) independently and in duplicate using the Critical Appraisal Skills Programme (CASP) Checklist [18]. Consensus between the two reviewers was required, and any discrepancies were adjudicated by a third party. No studies were weighted or excluded based on the appraisal results.

### Data management, analysis, and synthesis

Our synthesis follows the three-stage Thomas and Harden approach to inductive thematic synthesis [19]. We completed two steps of this process, as our primary aim was to produce descriptive themes of barriers and facilitators to guideline implementation across different health guidelines to then map on the COM-B components. After extracting the reported barriers and facilitators, two team members (CM and YB) created a codebook that was grouped into recurrent themes (e.g., resources, staffing issues). The two team members then independently and in duplicate coded each extracted barrier and facilitator with the themes from the code book. If new codes emerged, they were added iteratively to the code book and the barriers and facilitators were re-themed accordingly. The frequency of the themes was tallied as the number of times the theme was mentioned across the included articles. Finally, the themes were mapped onto the COM-B components of the Behaviour Change Wheel by the two team members independently and in duplicate. Based on a synthesis of 19 previously published behaviour change frameworks, the Behaviour Change Wheel provides tables that link the central COM-B components to potential knowledge translation intervention functions based on their expected effectiveness in relation to the barriers and facilitators. For example, if physical opportunity is a barrier, then training, restriction, environmental restructuring, and enablement are potential intervention functions. Potential knowledge translation intervention functions were listed with their associated barriers and facilitators and COM-B components. Any discrepancies between the two members were resolved by a third party. All data analysis and synthesis were performed in Microsoft Excel. Table 1 provide definitions for the COM-B components and knowledge translation intervention functions as outlined by the Behaviour Change Wheel.

**Table 1:** Definitions of the COM-B constructs and intervention functions as outlined by the Behaviour Change Wheel [7]

	Definition
COM-B construct	
Physical capability	Physical skill, strength, or stamina
Psychological capability	Knowledge or psychological skills, strength, or stamina to engage in the necessary mental processes
Physical opportunity	Opportunity afforded by the environment involving time, resources, locations, cues, physical affordance
Social opportunity	Opportunity afforded by the interpersonal influences, social cues and cultural norms that influence the way that we think about things
Reflective motivation	Reflective processes involving plans (self-conscious intentions) and evaluations (beliefs about what is good and bad)
Automatic motivation	Automatic processes involving emotional reactions, desires (wants and needs), impulses, inhibitions, drive states, and reflex responses
Intervention function	
Environmental restructuring	Changing the physical or social context
Restrictions	Using rules to reduce the opportunity to engage in the target behaviour (or to increase the target behaviour by reducing the opportunity to engage in competing behaviours)
Education	Increasing knowledge or understanding
Persuasion	Using communication to induce positive or negative feelings to stimulate action
Incentivisation	Creating an expectation of reward
Coercion	Creating an expectation of punishment or cost
Training	Imparting skills
Enablement	Increasing means/reducing barriers to increase capability (beyond education and training) or opportunity (beyond environmental restructuring)
Modeling	Provide an example for people to aspire to or imitate

### Assessing our confidence in the review findings

Two review authors (CM and PH) assessed the level of confidence for each finding using the GRADE-CERQual [20]. GRADE-CERQual assesses confidence in the evidence based on four key components: methodological limitations of included studies, coherence of the review findings, adequacy of the data contributing to a review finding, and relevance of the included studies to the review question. After assessing each of the four components, we made a judgement about the overall confidence in the evidence supporting the review finding and report it as high, moderate, low, or very low. The final assessment was based on consensus among the two review authors. All findings started as high confidence and were graded down if there were important concerns regarding any of the GRADE-CERQual components.

### Summary of qualitative findings table and evidence profile

We present summaries of the findings and our assessments of confidence in these findings in the Summary of qualitative findings table (Table 3). We present detailed descriptions of our confidence assessment in an Evidence Profile (Additional file 3).

### Review author reflexivity

The authors of this article are a multidisciplinary group of researchers and clinicians focused on geriatrics and improving care provision in LTC. They have engaged in several research studies in LTC including assessment of barriers and facilitators to implementation of practices, development of guidelines, knowledge translation, and randomized controlled trials. Since we have prior experience assessing barriers and facilitators in the LTC setting, some biases may exist as we may have preconceived ideas of what barriers and facilitators exist. Included studies that were conducted by one of the authors of the current paper were analyzed by two team members who were not authors of the included studies.

## Findings

### Results of the search

**Fig. 1:**
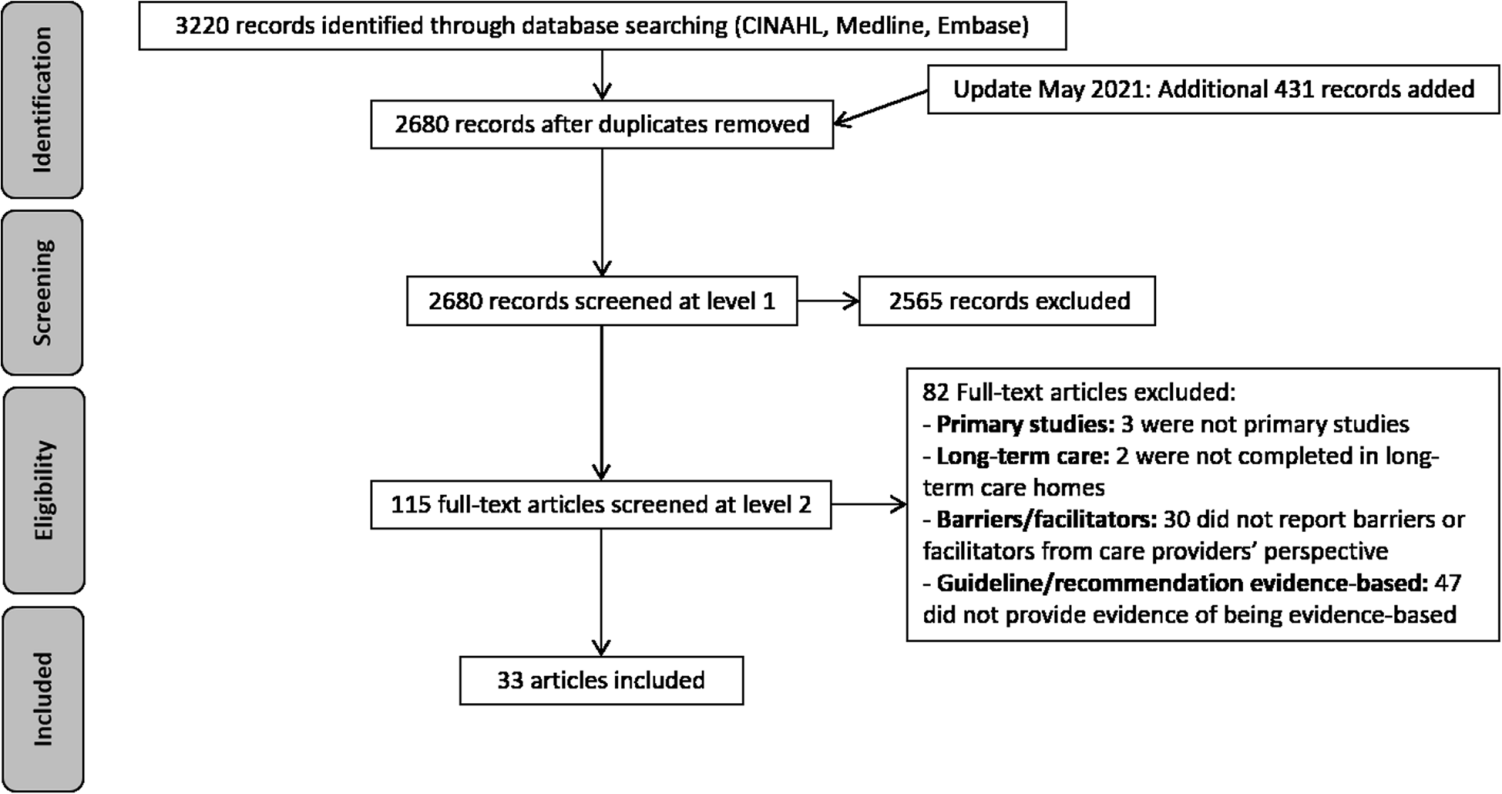
Flow of articles through the study

After screening 2680 articles, 33 that were published between 2004 and 2020 were included in the analyses (Fig. 1).

### Description of the studies

Most studies were conducted in Canada and Australia, with much fewer in the Netherlands, the USA, England, Sweden, Germany, South Korea, and Belgium (Table 2). A wide range of guidelines were examined, with the most frequent being oral health, medication reviews, and pain protocols. A variety of study designs were employed including qualitative studies, mixed method, multiple case studies, and process evaluations. Focus groups, interviews, and document analysis were the most frequent data collection methods, and thematic or content analysis was used to analyze data for 73% of included studies. Only six studies used a behaviour change framework, model, or theory to guide their work which included the framework developed by Greenhalgh et al. (Capability, Opportunity, and Motivation), Organizational Readiness for Change, Theoretical Domains Framework, Organization Learning Theory, Promoting Action in on Research Implementation in Health Services, and Normalization Process Theory.

**Table 2:** Characteristics of included studies

Study	Year	Country (Province, state, or region)	Study design	Study objectives	Data collection methods	Analysis	Guidelines and health topic examined	Behaviour change framework, model, or theory
Phipps et al. [36]	2019	England (South East)	Qualitative study	To explore what factors impact the ability of clinicians to manage care home flu outbreaks according to national guidelines and highlight opportunities for change	Collected notes written (from discussions) during responses to outbreaks, presentations on influenza at stakeholder engagement events	Identified and matched codes to themes—capability, opportunity, and organizational factors from framework	National guidelines supporting antiviral use	Framework developed by Greenhalgh et al., capability, opportunity, and motivation
Abraham et al. [37]	2019	Germany (Varied)	Process evaluation subcomponent of a pragmatic cluster randomized controlled trial	To systematically document the implementation process and describe barriers and facilitators	Structured interviews and focus groups	Not reported	IMPRINT—to reduce physical restraint use	None
Villarosa et al. [38]	2018	Australia (New South Wales)	Exploratory qualitative study	To explore the perceptions of care staff towards the provision of oral health care following implementation of a new care model: (1) examine the perceptions of the care staff regarding oral health care practices; (2) ascertain the needs and recommendations of care staff in relation to improving the delivery of oral health care.	Focus group	Inductive thematic analysis	Better oral health in Residential Aged Care programme	None
Huhtinen et al. [39]	2018	Australia (Sydney)	Mixed method	To identify the perceived barriers to the implementation of the Australian guidelines on influenza outbreak management with staff in an inner-city Sydney region	Telephone interview using a semi-structured questionnaire	Thematic analysis	National Guidelines for the Prevention, Control and Public Health Management of Influenza Outbreaks in Residential Care Facilities in Australia	None
Nilsen et al. [40]	2018	Sweden (Southern region)	Qualitative study	To identify barriers and facilitators to implementing evidence-based palliative care in the nursing homes	Semi-structured interviews	Content analysis. Codes were compared with categories outlined in the Organizational Readiness for Change Framework	World Health Organization guidelines on palliative care	Organizational Readiness for Change
DuBeau et al. [41]	2007	USA (Kansas)	Mixed method	To survey nursing home staff and state surveyors regarding attitudes about perceived and/or experienced barriers and challenges to implementing F315 compliance	Questionnaire survey with Likert type responses and open-ended questions	Inductive manifest and latent content analysis based on grounded theory	F315 tag: guidance for meeting compliance in managing and evaluating urinary incontinence and urinary catheters	None
Birney et al. [42]	2016	Canada (Alberta)	Exploratory qualitative study	To understand how four LTC facilities in Alberta have implemented medication reviews for the Appropriate Use of Antipsychotic Initiative	Semi-structured interviews and observations	Thematic analysis	Alberta Guideline on the Appropriate Use of Antipsychotic Medications	None
Fallon et al. [43]	2006	Australia (City of Toowoomba)	Quality improvement study	To identify barriers to implementation of evidence-based recommendations and strategies to overcome these barriers	Semi-structured focus group	Thematic analysis	Evidence-based recommendations for oral health	None
Baert et al. [44]	2016	Belgium (Flanders)	Mixed method	To identify barriers as well as motivators for organizing physical activity in LTC homes according to administrators on the different levels of the socioecological model. A secondary goal was examining their knowledge of the guidelines regarding physical activity and to reveal potential motivators and barriers for the implementation of these guidelines	Questionnaire and interviews	Deductive qualitative content analysis (Interviews)	World Health Organization guidelines for physical activity in older adults	None
Alamri et al. [45]	2015	Canada (Ontario)	Qualitative study	To identify potential barriers to evidence-based practices for osteoporosis and fracture prevention in LTC settings	Action plan worksheet completed by LTC staff in the control arm of an intervention study	Deductive and inductive thematic analysis	Clinical practice guidelines for the diagnosis and management of osteoporosis in Canada	Theoretical Domains Framework
Kaasalainen et al. [46]	2014	Canada (Ontario)	Exploratory, multiple-case study	What barriers and facilitators are encountered by the clinical nurse specialists and nurse practitioners in changing team practice related to implementing a pain protocol?	Diaries recording strategies, barriers, facilitators; participant observation and field notes by research assistant; interviews and focus groups	Thematic analysis	Interdisciplinary pain protocol by Kaasalainen et al. 2012	None
Vikstrom et al. [47]	2015	Sweden (Stockholm)	Implementation study	To outline the nursing home staff experiences during the first year of implementation of guidelines for care of people with dementia	Reflective seminars—detailed notes with experiential data relating to participant experiences in 4 discussions and written content/illustrations from posters	Inductive and deductive qualitative content analysis	Sweden's national evidence-based guidelines for care of people with dementia	None
Strachan et al. [48]	2014	Canada (Ontario)	Descriptive qualitative study nested in phase 2 of a three-phase mixed methods protocol	To explore LTC nurses’ experiences in managing heart failure	Focus group	Manifest content analysis	Canadian Cardiovascular Society Heart Failure guidelines in LTC	None
Lim et al. [49]	2014	Australia (Victoria)	Not reported	To explore the attitudes and perceptions of key healthcare providers towards antimicrobial stewardship interventions in Australian residential aged care facilities	Interviews and focus groups	Thematic analysis using the framework approach	International guidelines for infection control and prevention	None
Dellefield et al. [50]	2014	USA (California)	Exploratory qualitative interview	To describe nurses’ perceptions of individual and organization-level factors influencing performance of pressure ulcer prevention care in 2 VHA Nursing Home Community Living Centers to help identify existing factors perceived as facilitators and barriers to delivering pressure ulcer prevention care	Semi-structured interviews	Content analysis	Evidence-based guidelines for prevention of pressure ulcers	None
Berta et al. [28]	2013	Canada (Ontario)	Survey	To better understand how care protocols are implemented in LTC homes operating in Ontario, and to learn what processes, structural mechanisms, and knowledge sources are relevant to their implementation	Pen and paper survey	Mean score of importance	Clinical practice guidelines for either preventative skin care, wound/ulcer care, restraint use, management of incontinence, management of difficult behaviours, and antimicrobial resistance	Organizational learning theory
Bamford et al. [51]	2012	England (Not reported)	Process evaluation	To explore facilitators and barriers to the use of nutrition guidelines in residential care homes	Semi-structured interviews, informal discussions, nonparticipant observation	Thematic analysis, themes then mapped onto the Normalization Process Framework	UK Food Standards Agency nutrient and food-based guidance for older people in residential care	Normalization Process Theory
Kaasalainen et al. [52]	2012	Canada (Ontario)	Mixed method	To evaluate dissemination strategies in improving clinical practice behaviours (e.g., documentation of pain assessments, use of pain medications and non-pharmacological interventions) among health care team members, and the effectiveness of the pain protocol in reducing pain in LTC residents	Focus group and interviews	Thematic content analysis	The American Medical Directors’ Association and American Geriatrics Society best practice guidelines for pain	None
Verkaik et al. [53]	2011	Netherlands (Not reported)	Multiple case study	Which factors facilitate or inhibit successful introduction of the guideline in psychogeriatric nursing home wards? Which factors facilitate or inhibit the successful application of the guideline by CNAs in their support of residents with comorbid depression?	Semi-structured interviews, memos, evaluation forms, activity plan forms, training reports observations	Qualitative data analysis	Depression in Dementia	None
Berta et al. [54]	2010	Canada (Ontario)	Multiple case study	To explore the translational process that emerges within Ontario long-term care homes with the adoption and implementation of evidence-based clinical practice guidelines	Semi-structured interviews, focus groups	Template analysis via constant comparative analysis	Clinical practice guidelines for either preventative skin care, wound/ulcer care, restraint use, management of incontinence, management of difficult behaviours, and antimicrobial resistance	Organizational learning theory
McConigley et al. [55]	2008	Australia (Perth)	Qualitative study	Identify barriers and facilitators to guideline implementation and strengths that could assist in the implementation process	Focus groups and interviews	Thematic analysis	Australian Pain Society for residents in residential aged care facilities	None
Cheek et al. [56]	2004	Australia (South)	Descriptive/exploratory multimethod multilayered design	To investigate the factors that influence the implementation of best practice guidelines with respect to quality use of medicines in residential aged care facilities	Critical Incident Technique, focus groups, and nominal groups	Not reported	Nursing Guidelines for Medication Management in Nursing Homes and Hostels, Guidelines for Medical Care of Older Persons in Nursing Homes and Hostels, Best Practice Model for the Supply of Pharmacy Services to Residential Care Facilities	None
Hilton et al. [57]	2016	Australia (not reported)	Mixed method	To determine the views and experiences of nurses and care staff in residential care settings in relation to (a) implementing best practice oral care guidelines with residents of long-term care setting who have chronic disabling health conditions and (b) the barriers and facilitators to the implementation of common oral care practices included in clinical guidelines	Online survey and focus group	Thematic content analysis	Several oral care guidelines	None
Lau et al. [58]	2007	USA (Michigan)	Not reported	To examine the importance of work-related factors such as interprofessional communication, participation in decision making, and relationships among clinical staff members, for the adoption of guidelines in nursing homes	Semi-structured interviews	Thematic analysis	Federal guidelines on medication delivery CMS-mandated drug regimen review quality indicators, modified Beers criteria, and other practice guidelines, such as those issued by the American Medical Directors Association	None
Buss et al. [59]	2004	Netherlands (Limburg, Noord-Brabant)	Qualitative study	To elucidate the views and beliefs of health care workers (especially enrolled nurses) in Dutch nursing homes about pressure ulcer prevention and about issues related with pressure ulcer prevention	Interviews, written pressure prevention protocols	Thematic analysis	Dutch National Guidelines for Pressure Ulcer Prevention	None
Van der Maaden et al. [60]	2017	Netherlands (Not reported)	Process evaluation	To provide further understanding on the lack on an intervention effect in the cluster randomized trial.	Observation, interviews, survey	Content analysis	Practice guidelines for optimal symptom relief of pneumonia for residents with dementia	None
Kong et al. [61]	2021	South Korea (Seoul Special City, Gtyeonggi-do, Incheon Metropolitan City, Gangwon-do)	Qualitative descriptive study	To describe nursing home staff's perceptions of the barriers and needs in implementing care for people with dementia in Korean nursing homes	Semi-structured interviews	Qualitative content analysis	Person-centred dementia care	None
Jeong et al. [62]	2020	South Korea (Not reported)	Mixed methods study	To identify the barriers to implementation of a CPG perceived by healthcare professionals	Semi-structured interviews	Thematic analysis	Clinical practice guidelines for management of delirium	None
Eldh et al. [63]	2020	England, Ireland, Netherlands, Sweden (Not reported)	Cluster randomized controlled trial with embedded realist evaluation	To demonstrate the added and unique contribution observations made in comparison with survey and stakeholder interviews in a mixed method implementation study	Non-participant observations, survey, and interviews	Content analysis	Continence Management Guidelines	Promoting Action on Research Implementation in Health Services Framework
Cossette et al. [64]	2020	Canada (Quebec)	Prospective closed cohort supplemented by a development evaluation	To identify barriers and enablers in relation to the long-term integration of the OPUS-AP strategy in routine care	Semi-structured interviews	Semi-inductive thematic analysis	Appropriate use of anti-psychotics for behavioural and psychological symptoms of dementia	None
Surr et al. [65]	2020	England (West Yorkshire, Oxfordshire, South London)	Pragmatic cluster randomized controlled trial with a process evaluation	To examine the perceived barriers to and facilitators of intervention implementation, the mechanisms of impact and the perceived impacts from the perspective of mappers, expert mappers, managers, staff, residents and relatives	Semi-structured interviews	Framework analysis	Dementia Care Mapping	None
Desveaux et al. [66]	2019	Canada (Ontario)	Qualitative process evaluation	To examine whether, how, and why an academic detailing intervention could improve evidence uptake and (2) identify perceived changes that occurred to inform outcomes appropriate for quantitative evaluation.	Semi-structured interviews	Inductive approach within the framework method	Fall prevention guideline	None
Walker [67]	2019	Australia (Not reported)	Process evaluation	To report on process outcomes of the ViDAus study evaluating the feasibility of this multifaceted, interdisciplinary knowledge translation intervention for the implementation of vitamin D supplement use in residential aged care facilities	Unclear	Not reported	Vitamin D supplementation guidelines	Promoting Action on Research Implementation in Health Services Framework

*LTC* long-term care

Included studies recruited 12 to 500 LTC home staff from a variety of professions including nursing, medicine, management, rehabilitation (e.g., physical and occupational therapy), pharmacy, and food services (Table 3). Many studies did not report the age or sex of their participants. For those that did, the mean age of included staff ranged from 38 to 54 years, and the percentage of participants who were female ranged from 46% to 100%. Convenience and purposeful sampling were the most common methods of recruitment. At the LTC home level, the number of homes included ranged from 2 to 120, and the number of residents per home ranged from 40 to 251; though many studies did not report these values (11% did not report number of homes, 46% did not report number of residents per home). Similarly, more than half (58%) of the included studies did not report the ownership or rurality of the included homes.

**Table 3:** Characteristics of included participants and LTC homes

		Individual participant characteristics						LTC home characteristics			
Study	Year	Profession	Number	Age Mean (SD)	Sex % female	Sampling technique	Response rate	n of homes	n of residents in home	Ownership	Rurality
Phipps et al. [36]	2019	Partners from health protection, primary care, pharmacy, local authority, National Health Service	NR	NR	NR	NR	NR	NR	NR	NR	NR
Abraham et al. [37]	2019	Nursing home leaders, nominated key nurses, randomly selected nursing staff, relatives, legal guardians, home advisory board	NR	NR	NR	NR	NR	120	Varied	NR	NR
Villarosa et al. [38]	2018	Residential aged care staff	12	38 (15.5)	91.7%	Purposeful	NR	2	NR	Rural	Community-owned, not-for-profit
Huhtinen et al. [39]	2018	Registered nurses, director of nursing, facility manager, chief executive officer	28	NR	NR	Convenience	46%	28	Varied (41 to > 100 residents)	Urban	61% non-profit, 39% privately owned
Nilsen et al. [40]	2018	Nursing home managers	22	54 (SD not reported)	100%	Convenience	100%	22	Varied (32 to 110 staff)	NR	NR
DuBeau et al. [41]	2007	Nursing home staff (administrator, nursing director, nursing assistants, nurse practitioners, nursing consultants, medical staff) and surveyors	500	NR	NR	Convenience	85%	NR	68.6% were < 100 residents	58% rural	50% for profit, 37% not for profit, 12% government run
Birney et al. [42]	2016	Registered nurses, licensed practical nurses, health care aides, pharmacists, and facility managers/directors, care manager, best practice lead	18	NR	NR	Purposeful	NR	4	50–221 residents	75% urban	75% public
Fallon et al. [43]	2016	Facility staff and managers	NR	NR	NR	Convenience	NR	2	40–71 resident	Urban	Publicly funded
Baert et al. [44]	2016	LTC home administrators	Qual = 24 Quant = 127	Qual = males 49 (7), females 43 (11) Quant = males 50 (7), females 44 (8)	Qual = 46% Quant = 47%	Multistage stratified random	Qual—not reported; Quant—127/761	NR	NR	Urban and rural	Public and private
Alamri et al. [45]	2015	Medical director, director of care, administrator, consultant pharmacist, food services director, and other medical, nursing, and rehabilitation representatives	NR	NR	NR	NR	NR	12	Mean 114 (SD 57.0) residents	Urban and rural	92% for profit
Kaasalainen et al. [46]	2014	Clinical nursing specialist and nursing practitioners	28	NR	82%	Purposeful	NR	2	110–130 residents	NR	50% for profit
Vikstrom et al. [47]	2015	Nurse aides, registered nurses, physical and occupational therapists, managers	200	NR	NR	NR	NR	1	200 residents	Suburban	NR
Strachan et al. [48]	2014	Registered nurses, registered practical nurses, nurse practitioners	33	NR	NR	Convenience	NR	4	96–251 residents	Both	Public and private, profit and not for profit
Lim et al. [49]	2014	Registered nurses, general practitioners, pharmacists	61	Nurses—70.3% (> 40) GPs—10% (> 40) Pharmacists—66.7% (> 40)	78.7%	Purposive and snowball	NR	12	NR	NR	NR
Dellefield et al. [50]	2014	Registered nurses, licensed vocational nurses, nurses’ assistants	16	50 (SD not reported)	88%	Purposeful stratified	64%	2	NR	NR	NR
Berta et al. [28]	2013	Directors of care	392	NR	NR	Purposeful	72%	392	33% large (> 150 residents)	76% urban	43% chain owned, 19% not for profit
Bamford et al. [51]	2012	Cooks, managers, care staff	43	NR	NR	Maximum variation purposeful	NR	5	25–40 residents	Small towns and villages	Publicly funded
Kaasalainen et al. [52]	2012	Licensed nurses, personal support workers, administrator, directors of care, pharmacist, advanced practice nurse, physiotherapist	NR	NR	NR	NR	NR	4	NR	NR	NR
Verkaik et al. [53]	2011	Certified nursing assistants	20	NR	NR	Purposeful	20/109	9	NR	NR	NR
Berta et al. [54]	2010	Senior clinical, administrator, direct care staff	28	NR	NR	Stratified purposeful	NR	7	NR	NR	NR
McConigley et al. [55]	2008	Nurses, physiotherapists, occupational therapists, management staff, general practitioners	53	44 (8.5)	88%	Unclear	65%	5	60-245 residents	NR	NR
Cheek et al. [56]	2004	Registered nurse, enrolled nurse, manager, direct care worker, pharmacist, general practitioner, physiotherapist, speech therapist	33	NR	NR	Purposeful	NR	12	NR	NR	NR
Hilton et al. [57]	2016	Enrolled nurses	51	NR	NR	NR	NR	1	NR	NR	NR
Lau et al. [58]	2007	physicians, registered nurses, nurses’ aides, pharmacists	17	NR	NR	Purposeful	100%	4	NR	NR	NR
Buss et al. [59]	2004	Enrolled nurses, team leaders, head nurses, staff nurses, and physicians	18	NR	NR	Purposeful	100%	5	NR	NR	NR
Van der Maaden et al. [60]	2017	Physicians	14 interviews, 25 survey	Interviews: 47 years; survey: 21 years	71.4% interviews, 84% survey	Purposeful	NR	16	Mean 106 residents (range 30–189)	NR	NR
Kong et al. [61]	2020	Nurses, nursing assistants, care workers	24	40–69 years	100%	Convenience	54.5%	6	Medium or large (61–296)	Urban	4 private, 2 public
Jeong et al. [62]	2020	Managers, registered nurses, health assistants	10	NR	100%	Convenience	NR	2	NR	NR	NR
Eldh et al. [63]	2020	LTC staff	NR	NR	NR	NR	NR	24	NR	NR	NR
Cossette et al. [64]	2019	Nurses, manager, staff	10	NR	NR	Purposive	NR	5	NR	NR	NR
Surr et al. [65]	2020	Managers, mappers, other members of staff	67	NR	NR	Purposive	NR	18	Mix of medium and large	Mix of urban and rural	NR
Desveaux et al. [66]	2019	Administrative leaders, physicians, pharmacists, and direct care providers	29	NR	75.9%	Purposive	NR	13	NR	NR	NR
Walker [67]	2019	Key contact person from each facility—site manager, deputy manager, director or deputy director of nursing	NR	NR	NR	Convenience	NR	41	NR	NR	NR

*LTC* long-term care, *n* number, *NR* not reported

### Methodological limitations of the studies

Most studies had a clear research aim which was appropriately addressed through a qualitative research design. Likewise, most studies employed appropriate recruitment strategies and data were collected in a way that addressed the research question. In some studies, the description of data analysis techniques was limited. Overall, we found poor reporting of research reflexivity across most of the included studies. Details of the assessments of methodological limitations for individual studies are found in Additional file 4.

### Confidence in the review findings

We had moderate or high confidence in all but one of our review findings. Confidence was most often downgraded due to concerns with methodological limitations including a lack of discussion about credibility of qualitative findings and a lack of reflexivity. The data was almost always relevant as most studies examined our phenomena and population of interest. The full CERQual evidence profile can be found in Additional file 3.

### Review findings

The line-by-line thematic analysis of barriers and facilitators is found in Additional file 5. Table 4 provides a summary of the identified barrier and facilitator themes, their definitions and frequency, the articles contributing to the theme, and the CERQual assessment and explanation of confidence in the findings. The most frequently identified barriers and facilitators were consistent across guideline topics, while others were more specific to the content of the guideline. For example, nearly all articles identified time constraints and inadequate staffing (high confidence), and cost and lack of resources (high confidence) as barriers. However, guideline impracticality (high confidence) and taking a reactive approach (moderate confidence) were only identified in articles that discussed physical activity, influenza immunization, pneumonia treatment, and heart failure. In some instances, barriers and facilitators were opposites of each other, with barriers being actual and facilitators being perceived. For example, if time and money were an identified barrier, the staff perceived they could more easily implement the guideline with more time and resources (facilitator). However, some facilitators were also actual. For example, champions to promote implementation of the guidelines within the home was an actual facilitator in several articles.

**Table 4:** GRADE-CERQual summary of qualitative review findings table: barriers and facilitators of implementing evidence-based guidelines in long-term care

	Summary of review finding	Contributing articles	Frequency	CERQual Assessment of confidence in the evidence	Explanation of CERQual assessment
**Barriers**	*Time constraints and inadequate staffing*: lack of time or personnel to carry out tasks as indicated by the guideline	[36, 37, 39–44, 47, 49, 53, 55–57, 60, 61, 63–65]	32	High confidence	Minor concerns regarding methodological limitations, no or very minor concerns regarding coherence, adequacy, and relevance
	*Knowledge gaps:* inadequate training, expertise, or awareness of the targeted condition or guideline recommendations	[36–41, 43, 48, 51, 53, 55–57, 61, 62, 65, 67]	26	High confidence	Minor concerns regarding methodological limitations, no or very minor concerns regarding coherence, adequacy, and relevance
	*Cost and lack of resources:* inadequate financial and other resources (e.g., equipment) to carry out tasks as indicated by the guideline	[36–42, 44, 45, 48, 51, 56, 57, 62, 63, 65]	25	High confidence	Minor concerns regarding methodological limitations, no or very minor concerns regarding coherence, adequacy, and relevance
	*Lack of teamwork:* lack of cooperation and role coordination among the resident’s circle of care, including the LTC staff, family members, clinicians, and specialized health professionals	[36, 41, 42, 49, 51, 53, 55–59, 61, 63, 66, 67]	22	High confidence	Minor concerns regarding methodological limitations, no or very minor concerns regarding coherence, adequacy, and relevance
	*Lack of organizational support:* lack of impetus for guideline implementation from LTC home management.	[37, 38, 43, 44, 51, 53, 54, 56, 57, 60, 64, 65, 67]	20	High confidence	Minor concerns regarding methodological limitations, no or very minor concerns regarding coherence, adequacy, and relevance
	*Resident complexity:* complex comorbidities of LTC residents	[36, 37, 44, 50, 52, 53, 56, 57, 63, 67]	19	High confidence	Minor concerns regarding methodological limitations, no or very minor concerns regarding coherence, adequacy, and relevance
	*Compromised communication and information flow:* inadequate communication of relevant information between the resident, their family, staff, and/or allied health professions	[45, 48, 49, 51, 52, 54, 56, 58, 61, 66]	15	High confidence	Minor concerns regarding methodological limitations, no or very minor concerns regarding coherence, adequacy, and relevance
	*Staff turnover:* frequent change in staff	[37, 41, 43, 47, 52, 53, 56, 63, 65, 67]	15	High confidence	Minor concerns regarding methodological limitations, no or very minor concerns regarding coherence, adequacy, and relevance
	*Belief against the guideline:* distrust of the guideline’s recommendations and/or of its evidence base	[36, 37, 39, 44, 51, 52, 58–60, 67]	15	High confidence	Minor concerns regarding methodological limitations, no or very minor concerns regarding coherence, adequacy, and relevance
	*Conflict with clinical autonomy:* guideline recommendations conflict with health professional’s independence for clinical judgement	[36, 40, 46, 47, 49, 51, 53, 56, 58–60, 62]	13	High confidence	Minor concerns regarding methodological limitations, no or very minor concerns regarding coherence, adequacy, and relevance
	*Emotional responses to work and confidence in skills:* staff having lack of interest, negative attitude towards work, or low confidence in their ability to carry out guideline recommendation	[37, 40, 51, 56, 57, 59, 61, 62, 65]	12	Moderate confidence	Moderate concerns regarding methodological limitations, minor concerns regarding adequacy, and no or very minor concerns regarding coherence and relevance
	*Competing priorities:* staff burdened with too many tasks to place guideline adherence at high priority	[36, 38, 44, 46, 50, 52, 56, 57, 60, 63, 67]	12	High confidence	Minor concerns regarding methodological limitations, no or very minor concerns regarding coherence, adequacy, and relevance
	*Reluctance to change:* comfort with existing behaviour and resistance to developing new ones.	[37, 38, 40, 41, 43–46, 51, 52, 54, 65]	11	High confidence	Minor concerns regarding methodological limitations, no or very minor concerns regarding coherence, adequacy, and relevance
	*Inconsistent practices:* variations in practice between different health professionals in the LTC homes.	[45, 46, 49, 56, 57]	8	Moderate confidence	Moderate concerns regarding methodological limitations, minor concerns regarding adequacy, and no or very minor concerns regarding coherence and relevance
	*Moral distress:* guideline conflicts with resident/staff values or generate perception that the guideline will cause negative outcomes.	[36, 41, 48, 53, 56]	8	Moderate confidence	Moderate concerns regarding methodological limitations, minor concerns regarding adequacy, and no or very minor concerns regarding coherence and relevance
	*Guideline complexity and associated workload:* guideline creates additional workload to the staff due to the nature of its recommendations or complexity to process and understand the tasks	[36, 39, 41, 46, 56, 65]	8	Moderate confidence	Moderate concerns regarding methodological limitations, minor concerns regarding adequacy, and no or very minor concerns regarding coherence and relevance
	*Healthcare system structure:* inability to follow the guidelines due to the organizational structure of the healthcare system	[36, 51, 54, 56]	5	Moderate confidence	Moderate concerns regarding methodological limitations, minor concerns regarding adequacy, and no or very minor concerns regarding coherence and relevance
	*Simultaneous changes or change fatigue:* guideline introduces too many changes at once or staff are burdened with too many changes	[37, 53, 54]	4	Moderate confidence	Moderate concerns regarding adequacy, minor concerns regarding methodological limitations, and no or very minor concerns regarding coherence and relevance
	*Limited physical environment:* lack of appropriate physical infrastructure to carry out guideline recommendations	[39, 47, 56, 61]	4	Moderate confidence	Moderate concerns regarding methodological limitations and adequacy, no or very minor concerns regarding coherence and relevance
	*Conflicting guidelines:* guideline conflicts with another guideline on the same topic or current practice in the LTC homes	[36, 47, 56]		Moderate confidence	Moderate concerns regarding adequacy, minor concerns regarding methodological limitations, and no or very minor concerns regarding coherence and relevance
	*Impractical guideline:* guideline is not practical to the LTC setting	[44, 60]	2	High confidence, moderate confidence	Minor concerns regarding methodological limitations and adequacy, no or very minor concerns regarding coherence and relevance
	*Reactive approach:* responding to problems once they occur rather than focusing on prevention	[36, 48]	2	Moderate confidence, high confidence	Moderate concerns regarding adequacy, minor concerns regarding methodological limitations, and no or very minor concerns regarding coherence and relevance
	*Lack of noticeable improvement from guideline implementation*	[65, 67]	2	Moderate confidence	Moderate concerns regarding adequacy, minor concerns regarding methodological limitations, and no or very minor concerns regarding coherence and relevance
	*Leadership and champions:* LTC managers and leaders support the guideline implementation. Experienced champions are present to actively promote change and provide support to organizational members	[28, 37, 40, 44, 46, 49, 52–54, 57, 64, 65]	20	High confidence	Minor concerns regarding methodological limitations, no or very minor concerns regarding coherence, adequacy, and relevance
	*Well-designed strategies, protocols, and resources:* designing strategies, protocols, and tools that promote guideline uptake and minimize burden on the LTC system	[28, 38, 40, 44, 53, 55, 57, 64, 65, 67]	19	High confidence	Minor concerns regarding methodological limitations, no or very minor concerns regarding coherence, adequacy, and relevance
**Facilitators**	*Support and coordination among staff:* collaborative decision-making, clear role coordination, and encouragement among LTC staff	[28, 37, 42, 44, 49, 50, 57, 61, 64, 65, 67]	18	High confidence	Minor concerns regarding methodological limitations, no or very minor concerns regarding coherence, adequacy, and relevance
	*Adequate knowledge and education:* continuous education and training specific to the LTC context to ensure that the care team have the knowledge and skills to carry out guideline interventions	[37, 38, 46, 50, 52, 55, 57–59, 61, 63–65, 67]	16	High confidence	Minor concerns regarding methodological limitations, no or very minor concerns regarding coherence, adequacy, and relevance
	*Involving residents and families:* engaging residents and families in decision-making and education	[38, 42, 44, 50, 53, 57, 63, 65]	13	High confidence, high confidence	Minor concerns regarding methodological limitations, no or very minor concerns regarding coherence, adequacy, and relevance
	*Positive emotional responses to work and the intervention:* the resident’s care team value the intervention and demonstrate interest in developing care	[40, 52–54, 64, 65]	13	High confidence	Minor concerns regarding methodological limitations, no or very minor concerns regarding coherence, adequacy, and relevance
	*Adequate services, resources, and time:* staff have enough resources and time to carry out guideline interventions	[28, 44, 46, 49, 50, 54, 57, 64]	12	High confidence	Minor concerns regarding methodological limitations, no or very minor concerns regarding coherence, adequacy, and relevance
	*Noticeable outcomes from guideline implementation:* positive outcomes following guideline usage	[28, 37, 44, 47, 53, 64]	12	Moderate confidence	Moderate concerns regarding methodological limitations, minor concerns regarding adequacy, and no or very minor concerns regarding coherence and relevance
	*Good communication and information flow:* information regarding new protocols or resident assessment is communicated promptly and regularly to and among the resident’s care team	[42, 44, 50, 54, 55]	7	Moderate confidence	Moderate concerns regarding methodological limitations, no or very minor concerns regarding coherence, adequacy, and relevance
	*Conviction that the guideline is evidence-based and will demonstrate improvement:* the resident’s care team believe that the guideline is evidence-based and that guideline interventions will lead to positive outcomes	[44, 50]	5	Low confidence	Serious concerns regarding adequacy, minor concerns regarding methodological limitations, no or very minor concerns regarding coherence and relevance
	*Innovative environmental modifications:* innovative physical modification in the physical environment that promotes guideline usage	[38, 63, 67]	5	High confidence	Minor concerns regarding methodological limitations, no or very minor concerns regarding coherence, adequacy, and relevance

Physical and social opportunity were the COM-B components that the identified barriers and facilitators mapped onto most frequently (Table 5). Within physical and social opportunity, time constraints and inadequate staffing (high confidence), cost and lack of resources (high confidence), and lack of teamwork (high confidence) and organizational support (high confidence) were frequently reported barriers, while leadership and champions (high confidence), well designed strategies, protocols, and resources (high confidence), and adequate services, resources and time (high confidence) were frequent facilitators. Training, restriction, environmental restructuring, modelling, and enablement are knowledge translation intervention functions suggested by the Behaviour Change Wheel to overcome barriers associated with physical and social opportunity. The COM-B component of psychological capability represented knowledge gaps (high confidence) as a barrier and adequate knowledge and education (high confidence) as a facilitator. Education, training, environmental restructuring, modeling, and enablement are knowledge translation intervention functions suggested by the Behaviour Change Wheel to overcome barriers associated with psychological capability. Finally, reflective and automatic motivation had barriers relating to conflict with clinical autonomy (high confidence), beliefs against the guideline (high confidence), moral distress (moderate confidence), reluctance to change (high confidence), emotional responses to work and confidence in skills (moderate confidence), and change fatigue (moderate confidence). Facilitators with respect to reflective and automatic motivation were having noticeable outcomes occur from guidelines implementation (moderate confidence), a sense of conviction that the guidelines are evidence-based and will demonstrate improvement (low confidence), and a positive emotional response to work and the intervention (high confidence). The Behaviour Change Wheel suggests training, education, persuasion, modelling, enablement, incentivization, coercion, and environmental restructuring as potential knowledge translation interventions to overcome automatic and reflective motivation.

**Table 5:** Barrier and facilitator themes linked to COM-B constructs and Behaviour Change Wheel intervention functions

COM-B construct		Theme	Behaviour Change Wheel linked potential intervention functions
**Physical capability:** physical skill, strength, or stamina		None	None
**Psychological capability:** knowledge or psychological skills, strength or stamina to engage in the necessary mental processes	*Barriers*	Knowledge gaps	Education Training Environmental restructuring Modelling Enablement
	*Facilitators*	Adequate knowledge and education	
**Physical opportunity:** opportunity afforded by the environment involving time, resources, locations, cues, physical affordance		Time constraints and inadequate staffing	Training Restriction Environmental restructuring Enablement
	*Barriers*	Cost and lack of resources	
		Resident complexity	
		Compromised communication and information flow	
		Staff turnover	
		Competing priorities	
		Guideline complexity and associated workload	
		Healthcare system structure	
		Limited physical environment	
		Conflicting guidelines	
		Impractical guideline	
	*Facilitators*	Well-designed strategies, protocols, and resources	
		Adequate services, resources, and time	
		Innovative environmental modifications	
**Social opportunity:** opportunity afforded by the interpersonal influences, social cues and cultural norms that influence the way that we think about things	*Barriers*	Lack of teamwork	Restriction Environmental restructuring Modelling Enablement
		Lack of organizational support	
		Inconsistent practices	
		Reactive approach	
	*Facilitators*	Leadership and champions	
		Support and coordination among staff	
		Involving residents and families	
		Good communication and information flow	
**Reflective motivation:** reflective processes involving plans (self-conscious intentions) and evaluations (beliefs about what is good and bad)	*Barriers*	Conflict with clinical autonomy	Education Persuasion Modelling Enablement Incentivisation Coercion
		Belief against the guideline	
		Moral distress Lack of noticeable outcomes from guideline implementation	
	*Facilitators*	Noticeable outcomes from guideline implementation	
		Conviction that the guideline is evidence-based and will demonstrate improvement	
**Automatic motivation:** automatic processes involving emotional reactions, desires (wants and needs), impulses, inhibitions, drive states and reflex responses	*Barriers*	Reluctance to change	Training Incentivisation Coercion Environmental restructuring Persuasion Modelling Enablement
		Emotional responses to work and confidence in skills	
		Simultaneous changes or change fatigue	
	*Facilitators*	Positive emotional responses to work and the intervention	

### Review author reflexivity

We previously described our initial positioning earlier (see review author reflexivity above). Throughout the review, our positioning remained the same. During analysis and writing of the discussion, we felt our findings confirmed our initial ideas about the most frequent barriers and facilitators.

## Discussions

### Summary of the main findings

We systematically identified barriers and facilitators to implementing evidence-based guidelines in LTC and used behaviour change theory to link them to candidate knowledge translation functions. Across several guideline topics, time constraints and inadequate staffing, cost and lack of resources, knowledge gaps, and lack of teamwork and organizational support were frequently identified barriers. In contrast, leadership and champions, well-designed strategies, protocols, and resources, and adequate services, resources and time were frequently identified as facilitators. Linking to the central components of the Behaviour Change Wheel suggests physical and social opportunities and psychological capability are common targets for change to overcome barriers and leverage facilitators. While the most frequently identified barriers and facilitators appear to be universal regardless of guideline topics (e.g., pain, mood, physical activity, heart failure), some guidelines may have nuanced actions that have unique barriers and facilitators. We suggest that future knowledge translation and implementation science researchers assume the most frequently identified barriers and facilitators in our review are present and that they design strategies targeted at physical and social opportunity and psychological capability. A further analysis of barriers and facilitators may be necessary if the actions outlined by the guideline have unique features that could create additional barriers and facilitators.

The reported barriers and facilitators in our qualitative systematic review most frequently mapped onto the central Behaviour Change Wheel components physical and social opportunity: the opportunities afforded by the environment (e.g., time, resources, locations, cues, physical affordances) and interpersonal influences (e.g., social cues and cultural norms that influence the way we think about things). The findings that environmental opportunities (e.g., changing the social and physical context of care provision) are significant barriers to implementing evidence-based guidelines echo recent concerns surrounding quality of care provided in LTC highlighted by the COVID-19 pandemic [21] and is consistent with previous literature. Indeed, there have been recurrent reports of lack of funding and subsequent personnel shortages leading to decreased time to provide services to increasingly complex residents in LTC [22, 23]. Limited teamwork has also previously been identified as a barrier in LTC [24]. Linkage within the Behaviour Change Wheel suggests that training, restriction, environmental restructuring, enablement, and modelling are candidate knowledge translation intervention functions to overcome the identified barriers and leverage the facilitators.

Given the recent international interest in improving LTC during and after the COVID-19 pandemic and the subsequent impetus to support significant changes to the sector [21, 25], several of the Behaviour Change Wheel identified intervention functions could be feasible. For example, environmental restructuring involves changing the physical or social context to support guideline implementation. Resident-centred care approaches restructure the environment of care provision around the resident and address several of the barriers and facilitators identified in our review. For example, one such evidence-based approach, Neighbourhood Team Development, focuses on modifying the physical LTC environment, reorganizing delivery of care services, and aligning team members (e.g., LTC staff, family, residents) to collaborate in providing care [26]. Several of the studies included in our review also identified involving residents and family members as a facilitator of implementing evidence-based guidelines, supporting a resident-centred care approach.

Knowledge gaps pertaining to the information within guidelines, change fatigue, and lack of interest in work were frequently identified barriers and facilitators in our systematic review, which mapped onto the COM-B domains of psychological capability and reflective and automatic motivation. In many countries, most direct care within LTC homes is provided by care aides (e.g., personal support workers, health care aides, continuing care assistants, resident assistants) [27, 28] who often have the lowest level of education, receive the lowest financial compensation, have the least autonomy, and experience work-related burnout and poor job satisfaction [27, 29]. Knowledge gaps also apply to other members of the LTC interprofessional teams including licensed nurses, physicians, pharmacists, and rehabilitation and recreation and leisure providers. Indeed, several of the studies included in our review revealed knowledge gaps for different members of the LTC team. Education and training are potential knowledge translation intervention functions to overcome barriers associated with psychological capability and reflective and automatic motivation. Training for care aides is variable within and between countries. For example, in Canada, there are currently no national education standards for care aides working in LTC, and training varies widely between provinces [30]. Training of other members of the interprofessional team (e.g., physicians, physical therapists) often does not include a focus on geriatrics or LTC, nor is it standardized. Indeed, the COVID-19 pandemic revealed a major gap in standardized training for all team members about proper personal protective equipment use and conservation [31]. Consistent education and training with monitored national standards for all LTC staff may be one targeted knowledge translation strategy. However, for continuing education to be effective in LTC, it must be supported by the organization, and ongoing expert support is needed to enable and reinforce learning [32] which further bolsters the argument for a team-based, resident-centred approach.

### Comparison with other reviews and implications for the field

This is the first study to synthesize barriers and facilitators to guideline implementation in LTC from the perspectives of staff across healthcare conditions. Barriers and facilitators to guideline implementation have been systematically reviewed in other healthcare settings, but until now, no syntheses have been developed for the LTC context. Further, we not only identified the barriers and facilitators but also mapped them onto the central constructs of the Behaviour Change Wheel. This helps us explore the reasons why the factors identified are barriers and facilitators and the findings can be used to inform the development of future theory-guided knowledge translation intervention development.

### Overall completeness and applicability of the evidence

From a methodological point of view, the studies included in our review had several limitations. First, studies often did not report important information about the LTC home(s) which provides context from which the results were derived, such as the size, ownership, and rurality of the LTC home. The context of the LTC home including number of residents in a home, funding structure, and access to resources has been previously shown to affect implementation of best practice guidelines in LTC [14]. Future authors of LTC research are encouraged to fully describe the setting so that readers can adequately assess the generalizability of the results to their context, or reasons why they may experience different outcomes. Further, authors should include a fulsome description of the context including care philosophy of the home, staffing levels, and health system influences (e.g., public or private funding). Second, most authors did not critically examine their own role, potential bias, and influence during analysis and presentation of results. Reflexivity, or the acknowledgement of underlying beliefs and values held by researcher in selecting and justifying their methodological approach [33], is essential in assessing the authenticity of qualitative results [34]. Authors of qualitative research are encouraged to include a reflexive statement when reporting their results that describes their role in data collection, analysis and interpretation, and potential resulting biases that may arise.

### Limitations of the review

A strength of our study is that we synthesized information across different health conditions within the LTC sector. Given that there are likely many similarities among barriers and facilitators across guidelines for different conditions in the LTC setting, the findings of this qualitative evidence synthesis can help inform the implementation of any evidence-based guideline in LTC homes. However, a limitation of our study is that we did not assess the strength of the barriers and facilitators identified in this review. A frequently identified barrier may not hinder implementation as much as one that is less frequently reported. We argue that frequently reported barriers across several guideline topics are nonetheless important to identify as they can inform design of knowledge translation strategies regardless of topic. Future work should examine the strength of barriers and facilitators in LTC for implementing evidence-based guidelines and determine which barriers significantly limit implementation to add to our work. Another limitation is that we did not complete the third stage of the Thomas and Harden approach to thematic synthesis [19] to develop analytical themes that enable the development of new theoretical insights and findings not seen at individual primary study level. However, we saw mapping the barriers and facilitator themes onto the COM-B components as a way to take our analysis to the next step and provide recommendations for theory-guided knowledge translation strategies and understand why barriers and facilitators may exist. Additionally, as per the Thomas and Harden approach, we did not code directly onto any part of the manuscripts and focused our extraction on the results and findings sections, meaning key evidence may have been missed. We only included studies published in English which limits the generalizability of our findings to English-speaking countries or those that can pay for translation services. There is subjectivity in mapping of barriers and facilitators onto the COM-B components; some barriers and facilitators could map onto different components depending on the readers’ interpretations. Though we identified candidate intervention functions for implementing guidelines in LTC, we did not assess which ones are feasible and realistic to implement. Our next steps are to use the APEASE criteria [35] in consultation with stakeholders to determine the most appropriate intervention functions for the LTC sector.

## Conclusion and implications

### Implications for practice

We suggest that people designing LTC interventions to support guideline implementation assume the most frequently identified barriers (time constraints and inadequate staffing, cost and lack of resources, knowledge gaps, and lack of teamwork and organizational support) and facilitators (leadership and champions, well-designed strategies, protocols, and resources, and adequate services, resources and time) in our review are present and design strategies targeted at physical and social opportunity and psychological capability. Further analysis of barriers and facilitators specific to the guideline they are implementing may be necessary if the actions outlined by the guideline have unique features that could cause additional barriers and facilitators.

### Implications for research

Implications for research have been developed based on the findings of our study and our GRADE-CERQual assessment of findings. Future qualitative work in this area should transparently report researcher reflexivity including a reflection of the researchers’ roles and the influence this may have on the findings of the study. Additionally, researchers must fully describe the context of their LTC setting to ensure readers can determine whether the findings apply to their local LTC context. A full description of context would include the care philosophy of the home, staffing levels, and health system influences (e.g., public or private funding) among other factors.

## Supplementary Information

**Additional file 1.** ENTREQ Checklist.

**Additional file 2.** Search Strategy.

**Additional file 3.** Evidence Profile.

**Additional file 4.** CASP Checklist.

**Additional file 5.** Barriers and Facilitators Analysis.

## Data Availability

Not applicable.

## Abbreviations

COM-B: Capabilitiy, opportunity, motivation, behaviour
LTC: Long-term care
