# Supplementary material for: Barriers and facilitators to implementing evidence-based guidelines in long-term care: a qualitative evidence synthesis
Source: Implement Sci. 2021 Jul 9;16:70. doi: 10.1186/s13012-021-01140-0 (PMC8267230; doi:10.1186/s13012-021-01140-0)
Supplement: Supplementary file 2 — Additional file 2. Search Strategy. [file 13012_2021_1140_MOESM2_ESM.docx]

**MEDLINE** database searched through Ovid; includes articles 1946-present.

Limit to English only

|  |  |  |
| --- | --- | --- |
| 1 | **TITLE/ABSTRACT**  Homes for the age*  Home for the age*  Old age home*  Long term care  LTC  Nursing home  care home*  residential care facilit*  Residential aged care  Aged care facilit*  Geriatric facilit*  Geriatric care home*  elderly facilit*  **MESH**  Homes for the aged  Long-term care  Nursing homes  residential facilities  [Long-Term Care/organization & administration](https://www.ncbi.nlm.nih.gov/pubmed/25704127) | 71045 |
| 2 | **TITLE/ABSTRACT**  Guideline*  recommend*  protocol  **MESH**  Evidence-Based Practice*  Practice Guidelines as Topic*  Homes for the Aged/standards*  Nursing Homes/standards* | 1201857 |
| 3 | **TITLE/ABSTRACT**  Implement*  Application  Translation*  **MESH**  Translational Medical Research  Professional Practice | 1383709 |
| 4 | **TITLE/ABSTRACT**  Barrier*  Facilitat*  enable*  experience*  Attitude*  perception*  learning  challenge*  benefit*  Success*  constrain*  difficult*  enhanc*  Influen*  interfer*  motivat*  obstruct*  problem*  promot*  restrain*  restrict*  disincentive*  Factor* Capacity | 12395214 |
| 1+2 |  | 139112 |
| 1+2+3 |  | 98792 |
| 1+2+3+4 |  | 1096 |
