# Supplementary material for: Barriers and facilitators to implementing evidence-based guidelines in long-term care: a qualitative evidence synthesis
Source: Implement Sci. 2021 Jul 9;16:70. doi: 10.1186/s13012-021-01140-0 (PMC8267230; doi:10.1186/s13012-021-01140-0)
Supplement: Supplementary file 3 — Additional file 3. Evidence Profile. [file 13012_2021_1140_MOESM3_ESM.docx]

CERQual Evidence Profile

| Summary of review finding | Contributing articles | Methodological limitations | Coherence | Adequacy | Relevance | CERQual assessment of confidence in the evidence | Explanation of the CERQual assessment |
| --- | --- | --- | --- | --- | --- | --- | --- |
| *Time constraints and inadequate staffing:* Lack of time or personnel to carry out tasks as indicated by the guideline. | (36,37,39–44,47,49,53,55–57,60,61,63–65) | Minor concerns regarding methodological limitations due to lack of discussion by the primary authors about credibility of the results and reflexivity | No or very minor concerns regarding coherence | No or very minor concerns regarding adequacy | No or very minor concerns regarding relevance | High confidence | Minor concerns regarding methodological limitations, no or very minor concerns regarding coherence, adequacy, and relevance |
| *Cost and lack of resources:* Inadequate financial and other resources (e.g., equipment) to carry out tasks as indicated by the guideline. | (36–42,44,45,48,51,56,57,62,63,65) | Minor concerns regarding methodological limitations due to lack of discussion by the primary authors about credibility of the results and reflexivity | No or very minor concerns regarding coherence | No or very minor concerns regarding adequacy | No or very minor concerns regarding relevance | High confidence | Minor concerns regarding methodological limitations, no or very minor concerns regarding coherence, adequacy, and relevance |
| *Knowledge gaps:* Inadequate training, expertise, or awareness of the targeted condition or guideline recommendations. | (36–41,43,48,51,53,55–57,61,62,65,67) | Minor concerns regarding methodological limitations due to lack of discussion by the primary authors about credibility of the results and reflexivity | No or very minor concerns regarding coherence | No or very minor concerns regarding adequacy | No or very minor concerns regarding relevance | High confidence | Minor concerns regarding methodological limitations, no or very minor concerns regarding coherence, adequacy, and relevance |
| *Lack of teamwork:* Lack of cooperation and role coordination among the resident’s circle of care, including the LTC staff, family members, clinicians, and specialized health professionals. | (36,41,42,49,51,53,55–59,61,63,66,67) | Minor concerns regarding methodological limitations due to lack of discussion by the primary authors about credibility of the results and reflexivity | No or very minor concerns regarding coherence | No or very minor concerns regarding adequacy | No or very minor concerns regarding relevance | High confidence | Minor concerns regarding methodological limitations, no or very minor concerns regarding coherence, adequacy, and relevance |
| *Lack of organizational support:* Lack of impetus for guideline implementation from LTC home management. | (37,38,43,44,51,53,54,56,57,60,64,65,67) | Minor concerns regarding methodological limitations due to lack of discussion by the primary authors about credibility of the results and reflexivity | No or very minor concerns regarding coherence | No or very minor concerns regarding adequacy | No or very minor concerns regarding relevance | High confidence | Minor concerns regarding methodological limitations, no or very minor concerns regarding coherence, adequacy, and relevance |
| *Resident complexity:* Complex comorbidities of LTC residents. | (36,37,44,50,52,53,56,57,63,67) | Minor concerns regarding methodological limitations due to lack of discussion by the primary authors about credibility of the results and reflexivity | No or very minor concerns regarding coherence | No or very minor concerns regarding adequacy | No or very minor concerns regarding relevance | High confidence | Minor concerns regarding methodological limitations, no or very minor concerns regarding coherence, adequacy, and relevance |
| *Compromised communication and information flow:* Inadequate communication of relevant information between the resident, their family, staff, and/or allied health professions. | (45,48,49,51,52,54,56,58,61,66) | Minor concerns regarding methodological limitations due to lack of discussion by the primary authors about credibility of the results and reflexivity | No or very minor concerns regarding coherence | No or very minor concerns regarding adequacy | No or very minor concerns regarding relevance | High confidence | Minor concerns regarding methodological limitations, no or very minor concerns regarding coherence, adequacy, and relevance |
| *Staff turnover:* Frequent change in staff. | (37,41,43,47,52,53,56,63,65,67) | Minor concerns regarding methodological limitations due to lack of discussion by the primary authors about credibility of the results and reflexivity | No or very minor concerns regarding coherence | No or very minor concerns regarding adequacy | No or very minor concerns regarding relevance | High confidence | Minor concerns regarding methodological limitations, no or very minor concerns regarding coherence, adequacy, and relevance |
| *Conflict with clinical autonomy:* Guideline recommendations conflict with health professional’s independence for clinical judgement. | (36,40,46,47,49,51,53,56,58–60,62) | Minor concerns regarding methodological limitations due to lack of discussion by the primary authors about credibility of the results and reflexivity | No or very minor concerns regarding coherence | No or very minor concerns regarding adequacy | No or very minor concerns regarding relevance | High confidence | Minor concerns regarding methodological limitations, no or very minor concerns regarding coherence, adequacy, and relevance |
| *Belief against the guideline:* Distrust of the guideline’s recommendations and/or of its evidence base. | (36,37,39,44,51,52,58–60,67) | Minor concerns regarding methodological limitations due to lack of discussion by the primary authors about credibility of the results and reflexivity | No or very minor concerns regarding coherence | No or very minor concerns regarding adequacy | No or very minor concerns regarding relevance | High confidence | Minor concerns regarding methodological limitations, no or very minor concerns regarding coherence, adequacy, and relevance |
| *Competing priorities:* Staff burdened with too many tasks to place guideline adherence at high priority. | (36,38,44,46,50,52,56,57,60,63,67) | Minor concerns regarding methodological limitations due to lack of discussion by the primary authors about credibility of the results and reflexivity | No or very minor concerns regarding coherence | No or very minor concerns regarding adequacy | No or very minor concerns regarding relevance | High confidence | Minor concerns regarding methodological limitations, no or very minor concerns regarding coherence, adequacy, and relevance |
| *Reluctance to change:* Comfort with existing behaviour and resistance to developing new ones. | (37,38,40,41,43–46,51,52,54,65) | Minor concerns regarding methodological limitations due to lack of discussion by the primary authors about credibility of the results and reflexivity | No or very minor concerns regarding coherence | No or very minor concerns regarding adequacy | No or very minor concerns regarding relevance | High confidence | Minor concerns regarding methodological limitations, no or very minor concerns regarding coherence, adequacy, and relevance |
| *Emotional responses to work and confidence in skills:* Staff having lack of interest, negative attitude towards work, or low confidence in their ability to carry out guideline recommendation. | (37,40,51,56,57,59,61,62,65) | Moderate concerns regarding methodological limitations due to lack of discussion by the primary authors about credibility of the results and reflexivity, and lack of detailed description of data analysis methods. | No or very minor concerns regarding coherence | Minor concerns regarding adequacy of data as the quantity of the data was generally good, but the one process evaluation had limited richness and they did not report how many participants, so it was difficult to judge quantity. | No or very minor concerns regarding relevance | Moderate confidence | Moderate concerns regarding methodological limitations, minor concerns regarding adequacy, and no or very minor concerns regarding coherence and relevance |
| *Inconsistent practices:* Variations in practice between different health professionals in the LTC homes. | (45,46,49,56,57) | Moderate concerns regarding methodological limitations due to lack of discussion by the primary authors about credibility of the results and reflexivity, and lack of detailed description of data analysis methods. | No or very minor concerns regarding coherence | Minor concerns regarding adequacy of data as the richness of the data was generally good, but the one study did not report how many participants, so it was difficult to judge quantity. | No or very minor concerns regarding relevance | Moderate confidence | Moderate concerns regarding methodological limitations, minor concerns regarding adequacy, and no or very minor concerns regarding coherence and relevance |
| *Moral distress:* Guideline conflicts with resident/staff values or generate perception that the guideline will cause negative outcomes. | (36,41,48,53,56) | Moderate concerns regarding methodological limitations due to lack of discussion by the primary authors about credibility of the results and reflexivity, and lack of detailed description of data analysis methods. | No or very minor concerns regarding coherence | Minor concerns regarding adequacy of data as the richness of the data was generally good, but the one study did not report how much data were obtained, so it was difficult to judge quantity. | No or very minor concerns regarding relevance | Moderate confidence | Moderate concerns regarding methodological limitations, minor concerns regarding adequacy, and no or very minor concerns regarding coherence and relevance |
| *Guideline complexity and associated workload:* Guideline creates additional workload to the staff due to the nature of its recommendations or complexity to process and understand the tasks. | (36,39,41,46,56,65) | Moderate concerns regarding methodological limitations due to lack of discussion by the primary authors about credibility of the results and reflexivity, and lack of detailed description of data analysis methods. | No or very minor concerns regarding coherence | Minor concerns regarding adequacy of data as the richness of the data was generally good, but the one study did not report how much data were obtained, so it was difficult to judge quantity. | No or very minor concerns regarding relevance | Moderate confidence | Moderate concerns regarding methodological limitations, minor concerns regarding adequacy, and no or very minor concerns regarding coherence and relevance |
| *Healthcare system structure:* Inability to follow the guidelines due to the organizational structure of the healthcare system. | (36,51,54,56) | Moderate concerns regarding methodological limitations due to lack of discussion by the primary authors about credibility of the results and reflexivity, and lack of detailed description of data analysis methods. | No or very minor concerns regarding coherence | Minor concerns regarding adequacy of data as the richness of the data was generally good, but the one study did not report how much data were obtained, so it was difficult to judge quantity. | No or very minor concerns regarding relevance | Moderate confidence | Moderate concerns regarding methodological limitations, minor concerns regarding adequacy, and no or very minor concerns regarding coherence and relevance |
| *Simultaneous changes or change fatigue:* Guideline introduces too many changes at once or staff are burdened with too many changes. | (37,53,54) | Minor concerns regarding methodological limitations due to lack of discussion by the primary authors about credibility of the results and reflexivity | No or very minor concerns regarding coherence | Moderate concerns regarding adequacy of data as the quantity of the data was limited, with only 3 studies where one did not report how many participants and one did not report how much data were obtained. | No or very minor concerns regarding relevance | Moderate confidence | Moderate concerns regarding adequacy, minor concerns regarding methodological limitations, and no or very minor concerns regarding coherence and relevance |
| *Limited physical environment:* Lack of appropriate physical infrastructure to carry out guideline recommendations. | (39,47,56,61) | Moderate concerns regarding methodological limitations due to lack of discussion by the primary authors about credibility of the results and reflexivity, and lack of detailed description of data analysis methods. | No or very minor concerns regarding coherence | Moderate concerns regarding adequacy of data as the quantity of the data was limited, with only 3 studies where one did not report how many participants and how much data were obtained. | No or very minor concerns regarding relevance | Moderate confidence | Moderate concerns regarding methodological limitations and adequacy, no or very minor concerns regarding coherence and relevance |
| *Conflicting guidelines:* Guideline conflicts with another guideline on the same topic or current practice in the LTC homes. | (36,47,56) | Minor concerns regarding methodological limitations due to lack of discussion by the primary authors about credibility of the results and reflexivity | No or very minor concerns regarding coherence | Moderate concerns regarding adequacy of data as the quantity of the data was limited, with only 3 studies where one did not report how many participants and two did not report how much data were obtained. | No or very minor concerns regarding relevance | Moderate confidence | Moderate concerns regarding adequacy, minor concerns regarding methodological limitations, and no or very minor concerns regarding coherence and relevance |
| *Impractical guideline:* Guideline is not practical to the LTC setting. | (44,60) | Minor concerns regarding methodological limitations due to lack of discussion by the primary authors about credibility of the results and reflexivity | No or very minor concerns regarding coherence | Minor concerns regarding adequacy of data as the quantity of the data was limited, with only 2 studies though richness of the data was good. | No or very minor concerns regarding relevance | High confidence | Minor concerns regarding methodological limitations and adequacy, no or very minor concerns regarding coherence and relevance |
| *Reactive approach:* Responding to problems once they occur rather than focusing on prevention. | (36,48) | Minor concerns regarding methodological limitations due to lack of discussion by the primary authors about credibility of the results and reflexivity | No or very minor concerns regarding coherence | Moderate concerns regarding adequacy of data as the quantity of the data was limited, with only 2 studies where one did not how much data were obtained. | No or very minor concerns regarding relevance | Moderate confidence | Moderate concerns regarding adequacy, minor concerns regarding methodological limitations, and no or very minor concerns regarding coherence and relevance |
| *Lack of noticeable improvement from guideline implementation*: The guideline has no noticeable effects on the residents or home outcomes | (65,67) | Minor concerns regarding methodological limitations due to lack of discussion by the primary authors about credibility of the results and reflexivity | No or very minor concerns regarding coherence | Minor concerns regarding adequacy of data as the quantity of the data was limited, with only 2 studies though richness of the data was good | No or very minor concerns regarding relevance | High confidence | Minor concerns regarding methodological limitations and adequacy, no or very minor concerns regarding coherence and relevance |
| *Leadership and champions:* LTC managers and leaders support the guideline implementation. Experienced champions are present to actively promote change and provide support to organizational members. | (28,37,40,44,46,49,52–54,57,64,65) | Minor concerns regarding methodological limitations due to lack of discussion by the primary authors about credibility of the results and reflexivity | No or very minor concerns regarding coherence | No or very minor concerns regarding adequacy | No or very minor concerns regarding relevance | High confidence | Minor concerns regarding methodological limitations, no or very minor concerns regarding coherence, adequacy, and relevance |
| *Well designed strategies, protocols, and resources:* Designing strategies, protocols, and tools that promote guideline uptake and minimize burden on the LTC system. | (28,38,40,44,53,55,57,64,65,67) | Minor concerns regarding methodological limitations due to lack of discussion by the primary authors about credibility of the results and reflexivity | No or very minor concerns regarding coherence | No or very minor concerns regarding adequacy | No or very minor concerns regarding relevance | High confidence | Minor concerns regarding methodological limitations, no or very minor concerns regarding coherence, adequacy, and relevance |
| *Adequate knowledge and education:* Continuous education and training specific to the LTC context to ensure that the care team have the knowledge and skills to carry out guideline interventions. | (37,38,46,50,52,55,57–59,61,63–65,67) | Minor concerns regarding methodological limitations due to lack of discussion by the primary authors about credibility of the results and reflexivity | No or very minor concerns regarding coherence | No or very minor concerns regarding adequacy | No or very minor concerns regarding relevance | High confidence | Minor concerns regarding methodological limitations, no or very minor concerns regarding coherence, adequacy, and relevance |
| *Support and coordination among staff:* Collaborative decision-making, clear role coordination, and encouragement among LTC staff. | (28,37,42,44,49,50,57,61,64,65,67) | Minor concerns regarding methodological limitations due to lack of discussion by the primary authors about credibility of the results and reflexivity | No or very minor concerns regarding coherence | No or very minor concerns regarding adequacy | No or very minor concerns regarding relevance | High confidence | Minor concerns regarding methodological limitations, no or very minor concerns regarding coherence, adequacy, and relevance |
| *Adequate services, resources, and time:* Staff have enough resources and time to carry out guideline interventions. | (28,44,46,49,50,54,57,64) | Minor concerns regarding methodological limitations due to lack of discussion by the primary authors about credibility of the results and reflexivity | No or very minor concerns regarding coherence | No or very minor concerns regarding adequacy | No or very minor concerns regarding relevance | High confidence | Minor concerns regarding methodological limitations, no or very minor concerns regarding coherence, adequacy, and relevance |
| *Innovative environmental modifications:* Innovative physical modification in the physical environment that promotes guideline usage. | (38,63,67) | Minor concerns regarding methodological limitations due to lack of discussion by the primary authors about sample, reflexivity, and credibility of findings | No or very minor concerns regarding coherence | No or very minor concerns regarding adequacy of the data | No or very minor concerns regarding relevance | High confidence | Minor concerns regarding methodological limitations, no or very minor concerns regarding coherence, adequacy, and relevance |
| *Involving residents and families:* Engaging residents and families in decision-making and education. | (38,42,44,50,53,57,63,65) | Minor concerns regarding methodological limitations due to lack of discussion by the primary authors about credibility of the results and reflexivity | No or very minor concerns regarding coherence | No or very minor concerns regarding adequacy | No or very minor concerns regarding relevance | High confidence | Minor concerns regarding methodological limitations, no or very minor concerns regarding coherence, adequacy, and relevance |
| *Noticeable outcomes from guideline implementation:* Positive outcomes following guideline usage. | (28,37,44,47,53,64) | Moderate concerns regarding methodological limitations due to lack of discussion by the primary authors about credibility of the results and reflexivity, and lack of detailed description of data collection methods. | No or very minor concerns regarding coherence | Minor concerns regarding adequacy of data as the quantity of the data was limited, with 1 study not reporting how much data were obtained | No or very minor concerns regarding relevance | Moderate confidence | Moderate concerns regarding methodological limitations, minor concerns regarding adequacy, and no or very minor concerns regarding coherence and relevance |
| *Positive emotional responses to work and the intervention:* The resident’s care team value the intervention and demonstrate interest in developing care. | (40,52–54,64,65,68) | Minor concerns regarding methodological limitations due to lack of discussion by the primary authors about credibility of the results and reflexivity | No or very minor concerns regarding coherence | No or very minor concerns regarding adequacy | No or very minor concerns regarding relevance | High confidence | Minor concerns regarding methodological limitations, no or very minor concerns regarding coherence, adequacy, and relevance |
| *Good communication and information flow:* Information regarding new protocols or resident assessment is communicated promptly and regularly to and among the resident’s care team. | (42,44,50,54,55) | Moderate concerns regarding methodological limitations due to lack of discussion by the primary authors about credibility of the results and reflexivity, and lack of detailed description of data analysis methods. | No or very minor concerns regarding coherence | No or very minor concerns regarding adequacy | No or very minor concerns regarding relevance | Moderate confidence | Moderate concerns regarding methodological limitations, no or very minor concerns regarding coherence, adequacy, and relevance |
| *Conviction that the guideline is evidence-based and will demonstrate improvement:* The resident’s care team believe that the guideline is evidence-based and that guideline interventions will lead to positive outcomes. | (44,50) | Minor concerns regarding methodological limitations due to lack of discussion by the primary authors about credibility of the results and reflexivity | No or very minor concerns regarding coherence | Serious concerns regarding adequacy of the data as the quantity of the data was limited, with only 2 studies contributing to the review finding | No or very minor concerns regarding relevance | Low confidence | Serious concerns regarding adequacy, minor concerns regarding methodological limitations, no or very minor concerns regarding coherence and relevance |
