# Supplementary material for: Barriers and facilitators to implementing evidence-based guidelines in long-term care: a qualitative evidence synthesis
Source: Implement Sci. 2021 Jul 9;16:70. doi: 10.1186/s13012-021-01140-0 (PMC8267230; doi:10.1186/s13012-021-01140-0)
Supplement: Supplementary file 4 — Additional file 4. CASP Checklist. [file 13012_2021_1140_MOESM4_ESM.docx]

CASP Checklist (Yes, No, Can’t Tell)

|  | Section A: Are the results valid? | | | | | | Section B: What are the results? | | | Section C: Will the results help locally? |
| --- | --- | --- | --- | --- | --- | --- | --- | --- | --- | --- |
|  | 1. Was there a clear statement of the aims of the research? | 2. Is the qualitative methodology appropriate? | 3. Was the research design appropriate to address the aims of the research? | 4. Was the recruitment strategy appropriate to the aims of the research? | 5. Was the data collected in a way that addressed the research issue? | 6. Has the relationship between the researcher and participants been adequately considered? | 7. Have ethical issues been taken into consideration? | 8. Was the data analysis sufficiently rigorous? | 9. Is there a clear statement of findings? | 10. How valuable is the research? |
| Phipps 2019 | Yes | Yes | Yes | Yes | Yes | Yes | Can't tell | Yes | Yes | Yes |
| Abraham 2019 | Yes | Yes | Yes | Can't tell | Yes | Yes | Yes | No | Yes | Yes |
| Villarosa 2018 | Yes | Yes | Yes | Yes | Yes | Yes | Yes | Yes | Yes | Yes |
| Huhtinen 2019 | Yes | Yes | Yes | Yes | Yes | Yes | Yes | No | Yes | Yes |
| Nilsen 2018 | Yes | Yes | Yes | Yes | Yes | Yes | Yes | Yes | Yes | Yes |
| DuBeau 2007 | Yes | Yes | Yes | Yes | Yes | Can’t tell | Can't tell | Yes | Yes | Yes |
| Birney 2016 | Yes | Yes | yes | Yes |  | Yes | Yes | Yes | Yes | Yes |
| Fallon 2016 | Yes | Yes | Yes | Yes | Yes | Yes | Can't tell | Yes | Yes | Yes |
| Baert 2016 | Yes | Yes | Yes | Yes | Yes | Can't tell | Yes | Yes | Yes | Yes |
| Alamri 2015 | Yes | Yes | Yes | Yes | Yes | Yes | Yes | Yes | Yes | Yes |
| Kaasalainen 2014 | Yes | Yes | Yes | Yes | Yes | Can’t tell | Yes | Yes | Yes | Yes |
| Vikstrom 2015 | Yes | Yes | Yes | Yes | Yes | Can’t tell | Yes | Yes | Yes | Yes |
| Strachan 2014 | Yes | Yes | Yes | Yes | Yes | Can’t tell | Yes | Yes | Yes | Yes |
| Lim 2014 | Yes | Yes | Yes | Yes | Yes | Can’t tell | Yes | Yes | Yes | Yes |
| Dellefield 2014 | Yes | Yes | Yes | Yes | Yes | Can’t tell | Yes | Yes | Yes | Yes |
| Berta 2013 | Yes | Yes | Yes | Yes | Yes | Can’t tell | Yes | Yes | Yes | Yes |
| Bamford 2012 | Yes | Yes | Yes | Yes | Yes | Can’t tell | Yes | Yes | Yes | Yes |
| Kaasalainen 2012 | Yes | Yes | Yes | Yes | Yes | Can’t tell | Yes | Yes | Yes | Yes |
| Verkaik 2011 | Yes | Yes | Yes | Yes | Yes | Can’t tell | Yes | Yes | Yes | Yes |
| Bertaq 2010 | Yes | Yes | Yes | Yes | Yes | Can’t tell | Yes | Yes | Yes | Yes |
| MCConigley 2008 | Yes | Yes | Yes | Yes | Yes | Yes | Yes | Yes | Yes | Yes |
| Cheek 2004 | Yes | Yes | Yes | Yes | Yes | Can’t tell | Yes | Yes | Yes | Yes |
| Hilton 2016 | Yes | Yes | Yes | Yes | Yes | Can’t tell | Yes | Yes | Yes | Yes |
| Lau 2007 | Yes | Yes | Yes | Yes | Yes | Can’t tell | Yes | Yes | Yes | Yes |
| Buss 2004 | Yes | Yes | Yes | Yes | Yes | Can’t tell | Yes | Yes | Yes | Yes |
| Van der Maaden 2017 | Yes | Yes | Yes | Yes | Yes | Can’t tell | No | Yes | Yes | Yes |
| Kong 2020 | Yes | Yes | Yes | Yes | Yes | Can’t Tell | Yes | Yes | Yes | Yes |
| Jeong 2020 | Yes | Yes | Yes | Can’t Tell | Can’t Tell | No | Yes | Can’t Tell | Yes | Yes |
| Eldh 2020 | Yes | Yes | Yes | Yes | Yes | No | Yes | Yes | Yes | Yes |
| Cossette 2019 | Yes | Yes | Yes | Can’t Tell | Yes | No | No | Can’t Tell | Yes | Yes |
| Surr 2020 | Yes | Yes | Yes | Yes | Yes | No | Yes | Yes | Yes | Yes |
| Desveaux 2019 | Yes | Yes | Yes | Yes | Yes | No | Yes | Yes | Yes | Yes |
| Walker 2019 | Yes | Yes | Yes | Yes | Yes | No | Yes | Can’t Tell | Yes | Yes |
