# Supplementary material for: Barriers and facilitators to implementing evidence-based guidelines in long-term care: a qualitative evidence synthesis
Source: Implement Sci. 2021 Jul 9;16:70. doi: 10.1186/s13012-021-01140-0 (PMC8267230; doi:10.1186/s13012-021-01140-0)
Supplement: Supplementary file 5 — Additional file 5. Barriers and Facilitators Analysis. [file 13012_2021_1140_MOESM5_ESM.docx]

**Supplementary File – Barriers and Facilitators Analysis**

**Table 1. Barriers and associated themes**

|  | **Identified barriers** | **Theme** |
| --- | --- | --- |
| Phipps et al. (2019) | Competing clinical priorities | Competing priorities |
|  | Lack of resources and personnel | Cost and lack of resources |
|  | Guidelines are too complex to process and understand | Guideline complexity and associated workload |
|  | Debate over whose role it is to undertake public health actions such as providing prophylaxis | Lack of teamwork |
|  | Wanting confirmation of diagnosis first | Reactive approach |
|  | Disagreement on the strength of the evidence base behind guidelines on antivirals | Belief against the guideline |
|  | Disagreement that the patient has influenza | Conflict with clinical autonomy |
|  | Refusal to prescribe without recent renal function result | Conflicting guidelines |
|  | Many care home residents unable to express their choice about receiving antivirals | Resident complexity  Moral distress |
|  | Insufficient time to assess patients, gain consent, and prescribe antivirals | Time constraints and inadequate staffing |
|  | Poor understanding of the service delivery model | Knowledge gaps  Health care system structure |
|  | Difficulty accessing antiviral stock | Cost and lack of resources |
|  | Lack of clarity as to who will fund the prescriptions | Cost and lack of resources  Healthcare system structure |
|  | Contractual agreements for who is responsible for out of hours cover not communicated clearly | Cost and lack of resources  Healthcare system structure |
| Abraham et al. (2019) | Belief against guideline | Belief against the guideline |
|  | Apathy (uncritical attitude) of relatives re. physical restraints | Emotional responses to work and confidence in skills |
|  | Time constraint | Time constraints and inadequate staffing |
|  | Lack of care equipment | Cost and lack of resources |
|  | Leaders' negative attitude | Lack of organizational support |
|  | Simultaneous structural changes | Simultaneous changes / Change fatigue |
|  | Lack of support from nursing home leaders | Lack of organizational support |
|  | Negative experiences of restraint reduction (e.g., being held responsible for a fall) | Emotional responses to work and confidence in skills |
|  | Lack of knowledge | Knowledge gaps |
|  | Lack of interest and motivation to change daily routines | Reluctance to change |
|  | Different cultural background and therefore different attitudes towards restraints | Reluctance to change |
|  | Resignation of key nurses from the nursing home | Staff turnover |
|  | Staff fluctuation | Staff turnover |
|  | Inadequate staffing | Time constraints and inadequate staffing |
|  | Low reputation of key nurses by nursing staff | Lack of organizational support |
|  | Simultaneous structural changes such as implementation of electronic nursing documentations | Simultaneous changes / Change fatigue |
|  | Specific needs of residents with dementia | Resident complexity |
| Villarosa et al. (2018) | Not prioritizing oral health because misconception that treatment is not worthwhile (noncompliance from residents) | Knowledge gap |
|  | Oral care is not a priority for residents with dementia | Competing priorities |
|  | Reluctance from family members to provide access to dental services due to a lack of awareness regarding the importance of oral health | Reluctance to change  Knowledge gaps |
|  | Cost | Cost and lack of resources |
|  | Lack of knowledge and awareness among staff | Knowledge gaps |
|  | Inconsistent training | Lack of organizational support |
|  | Lack of accessibility of dental care in facilities | Cost and lack of resources |
| Huhtinen et al. (2019) | Skepticism towards staff vaccination | Belief against the guideline |
|  | Guidelines takes a lot of time and effort to read | Guideline complexity and associated workload  Time constraints and inadequate staffing |
|  | Lacking physical infrastructure to separate residents during outbreak | Limited physical environment |
|  | Lack of compliance with infection control recommendations by staff, likely due to lack of understanding or education | Knowledge gaps |
|  | Difficulties with preparing for and implementing medical interventions (e.g., High cost of antiviral medication and insufficient time to access it in time e.g., within 24 hours of a confirmed outbreak) | Cost and lack of resources  Time constraints and inadequate staffing |
| Nilsen et al. (2018) | Lack of competence and confidence regarding palliative care issues | Knowledge gaps  Emotional responses to work and confidence in skills |
|  | Belief in capability to address or handle death or dying persons to be able to develop care in nursing home | Emotional responses to work and confidence in skills |
|  | Staff's willingness and determination to develop care | Emotional responses to work and confidence in skills |
|  | Attitudes to work in general - interest/engagement | Emotional responses to work and confidence in skills |
|  | Resources - financial and personnel | Cost and lack of resources |
|  | Lack of time | Time constraints and inadequate staffing |
|  | Decisional latitude - managers' autonomy to make work-related decisions conducive to developing care | Conflict with clinical autonomy |
|  | Negative attitude towards changes at work | Reluctance to change |
| DuBeau et al. (2007) | Required documentation | Guideline complexity and associated workload |
|  | Staff turnover | Staff turnover |
|  | Physician cooperation | Lack of teamwork |
|  | Cost | Cost and lack of resources |
|  | Gaps in knowledge | Knowledge gaps |
|  | Inadequate staffing | Time constraints and inadequate staffing |
|  | Resistance to change | Reluctance to change |
|  | Inadequate medical input from physicians, advanced practice nurses, and medical directors | Lack of teamwork |
|  | Concern that pelvic examinations and catheterization as intrusive to resident dignity | Moral distress |
| Birney et al. (2016) | Staff availability and resources | Time constraints and inadequate staffing  Cost and lack of resources |
|  | Scheduling antipsychotic medication reviews | Time constraints and inadequate staffing |
|  | Lack of physician involvement | Lack of teamwork |
| Fallon et al. (2016) | Non-compliant patients | Reluctance to change |
|  | Training (difficulty scheduling due to time constraints, poor understanding of completing post-education questionnaire) | Time constraints and inadequate staffing  Knowledge gaps |
|  | Dietary issues - inability to effect recommendations because dietician was responsible for dietary and sugar intake and was not a project team member | Staff turnover  Time constraints and inadequate staffing |
|  | Inadequate staffing/staff turnover | Staff turnover  Time constraints and inadequate staffing |
|  | Lack of organizational support | Lack of organizational support |
| Baert et al. (2016) | The guidelines are not feasible for residents (eg, PA is too intensive, frequency too high) | Impractical guideline |
|  | The residents are too old | Resident complexity |
|  | The residents are not interested in changing their health behavior | Reluctance to change |
|  | The poor physical condition of the residents | Resident complexity |
|  | These guidelines are going to have a negative impact on the  psychological well-being of the residents | Belief against the guideline |
|  | These guidelines are not going to have a positive impact on the general well-being of the residents | Belief against the guideline |
|  | Residents will demotivate each other | Belief against the guideline |
|  | Lack of time | Time constraints and inadequate staffing |
|  | Other topics are more urgent (eg, recruitment of new nurses, administrative work) | Competing priorities |
|  | Lack of material | Cost and lack of resources |
|  | Lack of infrastructure | Cost and lack of resources |
|  | Lack of staff | Time constraints and inadequate staffing |
|  | Lack of financial resources | Cost and lack of resources |
|  | These guidelines are optional | Lack of organizational support |
| Alamri et al. (2015) | Lack of information and educational resources for patient and staff | Cost and lack of resources |
|  | Few staff attend educational sessions | Cost and lack of resources |
|  | Difficulty acquiring necessary patient information for fracture risk assessment | Compromised communication and information flow |
|  | Inconsistent prescribing of vitamin D and calcium at the time of admission | Inconsistent practices |
|  | Cost of medication / Patients or his/her family not willing to pay for vitamin D | Cost and lack of resources |
|  | Guideline not included in multidisciplinary conferences and quarterly reviews | Compromised communication and information flow |
|  | Process to change is cumbersome | Reluctance to change |
|  | The LTC facility does not examine changes in height on an annual basis | Inconsistent practices |
|  | The recommendation of three servings of dairy per day is not followed | Inconsistent practices |
|  | Staff members incorrectly dispense bisphosphates | Inconsistent practices |
| Kaasalainen et al. (2014) | Lack of registered nurse follow-through | Inconsistent practices |
|  | Belief that protocol is too rigid | Conflict with clinical autonomy |
|  | Increased and more complicated paperwork | Guideline complexity and associated workload |
|  | Perception of low priority by staff | Competing priorities |
|  | Heavy workload and competing demands for staff | Competing priorities |
|  | Staff resistance to change | Reluctance to change |
|  | Staff turnover | Staff turnover |
| Vikstrom et al. (2015) | High workload | Time constraints and inadequate staffing |
|  | Lack of time | Time constraints and inadequate staffing |
|  | Low staffing levels | Time constraints and inadequate staffing |
|  | Lack of continuity of care | Staff turnover |
|  | Discrepancy between nation guidelines and local policies | Conflicting guidelines |
|  | Structure too rigid | Conflict with clinical autonomy |
|  | Architectural limitations in the physical environment | Limited physical environment |
| Strachan et al. (2014) | Reactive vs proactive approach | Reactive approach |
|  | Ability to interpret signs, symptoms and acuity | Knowledge gaps |
|  | Compromised information flow | Compromised communication and information flow |
|  | Moral distress | Moral distress |
|  | Access to resources | Cost and lack of resources |
| Lim et al. (2014) | Doctor autonomy hindering acceptance of institutional policies/guidelines | Conflict with clinical autonomy |
|  | Heterogeneity in prescribing practices of doctors | Inconsistent practices |
|  | High workload | Time constraints and inadequate staffing |
|  | Doctor acceptance | Conflict with clinical autonomy |
|  | Pharmacists’ on-site availability | Lack of teamwork |
|  | Inadequate access to patients' clinical information | Compromised communication and information flow |
|  | Limited communication with doctors and pharmacists | Lack of teamwork  Compromised communication and information flow |
| Dellefield et al. (2014) | Balancing tasks of inspecting skin with other on the job responsibilities or cognitive status | Competing priorities |
|  | Patients' weight or cognitive status | Resident complexity |
| Bamford et al. (2012) | Contesting the value of external guidelines | Acceptance of the guidelines |
|  | Incompatibility with existing goals | Conflicting guidelines |
|  | Questioning the benefits of dietary change for older people | Belief against the guideline |
|  | Satisfaction with existing menus | Reluctance to change |
|  | Perceived threats to autonomy and expertise | Conflict with clinical autonomy |
|  | Lack of focus or impetus for implementation | Lack of organizational support |
|  | Limited knowledge of nutritional content of food | Knowledge gaps |
|  | Lack of resources for implementation | Cost and lack of resources |
|  | Complex and unreliable procurement systems | Healthcare system structure |
|  | Lack of monitoring of implementation | Lack of organizational support |
|  | Lack of systematic feedback on the impacts on client well-being | Compromised communication and information flow |
|  | Concerns over the reliability of feedback mediated by care staff | Lack of teamwork |
|  | Lack of confidence in modifying menus and recipes | Emotional responses to work and confidence in skills |
| Kaasalainen et al. (2012) | Ongoing challenges assessing and treating pain in older adults | Resident complexity |
|  | Competing demands and priorities for nursing staff | Competing priorities |
|  | Personal values and beliefs about pain | Belief against the guideline |
|  | Initial resistance to change | Reluctance to change |
|  | Lack of interdisciplinary communication (both written and verbal) about residents' pain | Compromised communication and information flow |
| Verkaik et al. (2011) | Reorganizations or other innovations at the time of the guideline introduction | Simultaneous changes / Change fatigue |
|  | Top down introduction of the guideline | Conflict with clinical autonomy |
|  | Team manager does not recognize relevance of guideline introduction | Lack of organizational support |
|  | Social opportunity | Staff turnover |
|  | Shortage of certified personnel | Time constraints and inadequate staffing |
|  | Not sharing an emotion-oriented care vision | Lack of teamwork |
|  | View that depressed residents should not have more privileges than non-depressed residents | Moral distress |
|  | Expectation that guideline and training would bring instant solutions for depression in dementia | Knowledge gaps |
|  | Non-certified or non-registered nurses or nursing assistants were not trained | Knowledge gaps |
|  | View that a resident has the right to be depressed, | Moral distress |
|  | Employment: small contracts and alternating shifts | Staff turnover |
|  | Stage of dementia > 6 | Resident complexity |
|  | Multiple behavioural and emotional disturbances | Resident complexity |
|  | Feeling sick/having much pain | Resident complexity |
| Berta et al. (2010) | Absence of a learning culture | Lack organizational support |
|  | Entrenched ways of learning and communication | Reluctance to change  Compromised communication and information flow |
|  | Change fatigue | Simultaneous changes / Change fatigue |
|  | Union environment | Healthcare system structure |
| McConigley et al. (2008) | Guidelines demand more time than was feasible with current staff levels | Time constraints and inadequate staffing |
|  | Team meetings were difficult to achieve because of time | Time constraints and inadequate staffing |
|  | Expertise to implement some guidelines were lacking | Knowledge gaps |
|  | Limited availability of pain specialists because of time and lack of specialists | Lack of teamwork |
|  | Any guideline that required the completion of extra documentation would not be well received by staff because of time constraints | Time constraints and inadequate staffing |
|  | Care workers don't have skills and knowledge relevant to pain management | Knowledge gaps |
| Cheek et al. (2004) | Contextual/structural: Lack of continuity of staff and service providers, Skill mix/resources | Staff turnover  Knowledge gaps |
|  | Time: expectations of self/organisation, Management support, Lack of | Lack of organizational support  Time constraints and inadequate staffing |
|  | Commonwealth regulation | Healthcare system structure |
|  | Physical environment: facility layout | Limited physical environment |
|  | Boundaries pertaining to roles, blurring of boundaries, e.g. pharmacists; doctors; nurses registered nurses and enrolled nurses; careworkers | Lack of teamwork |
|  | The need for teamwork across boundaries | Lack of teamwork |
|  | Trust | Emotional responses to work and confidence in skills |
|  | Information flow | Compromised communication and information flow |
|  | Boundaries pertaining to decision making | Conflict with clinical autonomy  Lack of teamwork |
|  | Knowing where one stands – legal, responsibility/accountability | Lack of organizational support |
|  | Knowing – the resident, the doctor, the pharmacist, your staff, Balancing need and available resources, Role of the family, Personal/public boundaries, Home vs residential aged care | Lack of teamwork  Lack of organizational support |
|  | Independence vs dependence: who needs to know what? | Lack of teamwork  Lack of organizational support |
|  | Ethical: business vs service delivery | Moral distress  Health care system structure |
|  | Advocacy – wanting to do the right thing by the residents, providing a 24-hour service, being on call, trying to meet multiple demands | Competing priorities  Moral distress |
|  | Day-to-day practices: keeping the system going, complexity of communication, Problematic medication chart, being attached to the medication trolley – the medication round | Compromised communication and information flow |
|  | Monitoring: the system; medication outcomes; outstanding prescriptions | Compromised communication and information flow |
|  | Keeping Up: knowledge/education or lack of, knowing limitations and accessing information, | Knowledge gaps |
|  | Complexity of medications, Changing needs of residents | Resident complexity |
|  | Interruptions to nursing staff when administering | Time constraints and inadequate staffing |
|  | the need for all to continually chase up facets pertaining to medication use in the RACF | Cost and lack of resources |
|  | inflexible work practices | Lack of organizational support |
|  | a plethora of documentation which makes it hard to provides a summary of the subthemes that fall under track or provide information | Guideline complexity and associated workload |
|  | lack of standardised procedures with respect to medication-related practices | Inconsistent practices |
|  | untrained or lack of qualified staff | Time constraints and inadequate staffing  Knowledge gaps |
| Hilton et al. (2016) | Lack of equipment/product | Cost and lack of resources |
|  | Lack of funding for adequate oral care | Cost and lack of resources |
|  | Lack of family support for oral care | Lack of teamwork |
|  | Resident having fixed teeth | Resident complexity |
|  | Resident having loose dentures | Resident complexity |
|  | Resident behaviours | Resident complexity |
|  | Resident dependency/participation | Resident complexity |
|  | Resident having dysphagia | Resident complexity |
|  | Oral care is viewed as a low priority | Competing priorities |
|  | Lack of time | Time constraints and inadequate staffing |
|  | Negative attitude of staff | Emotional responses to work and confidence in skills |
|  | Inadequate staffing | Time constraints and inadequate staffing |
|  | Lack of staff education/training | Knowledge gaps |
|  | Systemic errors | Inconsistent practices |
|  | Lack of oral care policy education/enforcement | Lack of organizational support |
| Lau et al. (2007) | Guidelines must be grounded in validated scientific evidence for them to be applicable to individual clinical situations | Belief against the guideline |
|  | Guidelines could be viewed as rules imposed by the government that infringe upon professional autonomy and create additional work burden | Conflict with clinical autonomy |
|  | Hierarchy of roles and responsibilities among clinicians | Lack of teamwork |
|  | Conflicts among the care team members clearly put stress on their working relationships | Lack of teamwork |
|  | Lack of communication | Compromised communication and information flow |
| Buss et al. (2004) | Traditional knowledge, evidence-based knowledge | Belief against the guideline |
|  | Acceptance - personal involvement | Emotional responses to work and confidence in skills |
|  | Involvement of the team | Lack of teamwork |
|  | Conviction about effectiveness of intervention | Belief against the guideline |
|  | Utilization - having a protocol but not really following it | Conflict with clinical autonomy |
| van der Maaden et al. (2017) | Working already according to the guideline | Conflict with clinical autonomy |
|  | The information in the guideline is not new or innovative | Belief against the guideline |
|  | Hectic pace of daily practice in nursing homes | Competing priorities |
|  | Need to refamiliarize with guideline but lack of time to do so | Time constraints and inadequate staffing |
|  | Practical use of the guideline (e.g., cannot browse through while with a patient) | Impractical guideline |
|  | Fixed routines | Lack of organizational support |
| Kong et al. (2020) | Insufficient staff | Time constraints and inadequate staffing |
|  | Inappropriate physical environment | Limited physical environment |
|  | Staff’s lack of education | Knowledge gaps |
|  | Family’s lack of education | Knowledge gaps |
|  | Staff’s negative attitudes | Emotional response to work and confidence in skills |
|  | Staff’s hurtful experiences | Emotional response to work and confidence in skills |
|  | Lack of communication among staff, residents, and families | Compromised communication and information flow |
|  | Lack of trust between staff and families | Emotional response to work and confidence in skills |
|  | Conflicts among/between staff and families | Lack of teamwork |
| Jeong et al. 2020 | Lack of resources | Cost and lack of resources |
|  | Tendency to follow mindlines rather than guidelines | Conflict with clinical autonomy |
|  | Healthcare professionals’ passive attitude (this is not our job) | Emotional response to work and confidence in skills |
|  | Patient/family misunderstanding about delirium care in LTC | Knowledge gaps |
| Eldh et al. (2020) | Resources – lack of supplies | Cost and lack of resources |
|  | Staff-centredness | Competing priorities |
|  | Availability of staff | Time constraints and inadequate staffing |
|  | Unexpected needs of residents | Resident complexity |
|  | Lack of collaboration | Lack of teamwork |
|  | Staff turnover | Staff turnover |
| Cossette et al. (2019) | Resources | Lack of time and resources |
|  | Staff/work organization | Lack of organizational support |
| Surr et al. (2020) | Staffing levels | Time constraints and inadequate staffing |
|  | Staff turnover | Staff turnover |
|  | Availability of resources and funds | Cost and lack of resources |
|  | High workload intensity associated with the guideline | Guideline complexity and associated workload |
|  | Lack of support from managers | Lack of organizational support |
|  | Time constraints | Time constraints and inadequate staffing |
|  | Negative attitudes towards intervention | Emotional responses to work and confidence in skills |
|  | Lack of knowledge | Knowledge gaps |
|  | Complexity of guidelines | Guideline complexity and associated workload |
|  | Difficulties in measuring impacts for people with dementia | Lack of noticeable improvement from guideline implementation |
|  | Not being open to change | Reluctance to change |
| Desveaux et al. (2019) | Communication gaps | Compromised communication and information flow |
|  | Misaligned role expectations | Lack of teamwork |
|  | Being reactive | Reactive approach |
|  | Lack in teamwork | Lack of teamwork |
| Walker et al. (2019) | Residents’ and family beliefs or attitudes (polypharmacy, cost, cultural barriers) | Belief against the guideline |
|  | GP beliefs & attitudes | Belief against the guideline |
|  | Suitability of residents for vitamin D | Resident complexity |
|  | Competing priorities/time/capacity to implement | Competing priorities |
|  | Residents’ and family knowledge or understanding | Knowledge gaps |
|  | Awareness/process/prompt for staff | Knowledge gaps |
|  | Resident behaviours (medication refusal due to dementia, swallowing difficulties, or mental health) | Resident complexity |
|  | Contact with general practitioners (having many GPs or GPs that do not regularly visit) | Lack of teamwork |
|  | Staff turnover | Staff turnover |
|  | Resident turnover | Resident complexity |
|  | Leadership/culture/motivation to change | Lack of organizational support |
|  | GP knowledge | Knowledge gaps |
|  | Pharmacist attitudes & beliefs | Belief against the guideline |
|  | Staff knowledge | Knowledge gaps |
|  | No obvious way to demonstrate the benefits of vitamin D | Lack of noticeable improvement from guideline implementation |

Table 2. Facilitators and associated themes

|  | **Identified facilitators** | **Theme** |
| --- | --- | --- |
| Abraham et al. (2019) | Leader’s support | Leadership and support/champion |
|  | Mutual support and coordination between key nurses | Support and coordination among staff |
|  | Positive experiences (e.g. successful prevention of physical restraints) | Noticeable outcomes from guideline implementation |
|  | Continuous flow of information | Good communication and information flow |
|  | Positive attitudes toward the intervention among nursing staff | Positive emotional responses to work and the intervention |
|  | Adequate knowledge and continuous education about restraints | Adequate education / training |
|  | Teamwork | Support and coordination among staff |
|  | Willingness to take responsibility in decision-making and clinical practice to avoid/reduce physical restraints | Positive emotional responses to work and the intervention |
| Villarosa et al. (2018) | Establishing an onsite dental chair | Innovative environmental modifications |
|  | Mobile services | Innovative environmental modifications |
|  | Dental students could complete placements for residents to receive more comprehensive assessments | Well-designed strategies, protocols, and resources |
|  | Increasing frequency of oral health training including how to work with residents who are non-compliant (e.g., with dementia) | Adequate education/training |
|  | Raising oral health awareness - ensuring staff, residents, and families understand the importance of oral care | Involving residents and families  Adequate education/training |
| Nilsen et al. (2018) | Competence and confidence | Positive emotional responses to work and the intervention |
|  | Motivation – willingness and determination to develop care | Positive emotional responses to work and the intervention |
|  | Attitudes to changes in work – staff’s resolve to pursue development of care despite experiencing concurrent changes | Positive emotional responses to work and the intervention |
|  | Attitudes to work in general - interest/engagement | Positive emotional responses to work and the intervention |
|  | Plans for developing structures or concrete strategies for continued efforts to develop care | Well-designed strategies, protocols, and resources |
|  | Decisional latitude - managers' autonomy to make work-related decisions conducive to developing care | Leadership and support/champion |
|  | Leadership - influence of managers and other leaders on the staff | Leadership and support/champion |
| Birney et al. (2016) | Patient/Family-centred care | Involving residents and families |
|  | Collaborative decision-making | Support and coordination among staff |
|  | Role clarity | Support and coordination among staff |
|  | Interprofessional communication | Good communication and information flow |
|  | Interprofessional conflict resolution | Support and coordination among staff |
| Baert et al. (2016) | Desire to see improvement of LTCF residents’ functional status, self-sustainability, self-esteem, and quality of life | Conviction that the guideline is evidence-based and will demonstrate improvement |
|  | Conviction of the evidence-base of PA | Conviction that the guideline is evidence-based and will demonstrate improvement |
|  | Conviction that PA is important and beneficial to physical and psychological health | Conviction that the guideline is evidence-based and will demonstrate improvement |
|  | Organizing PA brings enjoyment/pleasure to the LTCF residents and their family | Noticeable outcomes from guideline implementation |
|  | Enhancing their own job satisfaction through organizing PA | Noticeable outcomes from guideline implementation |
|  | Social interaction during PA sessions, decreased loneliness | Noticeable outcomes from guideline implementation |
|  | The staff encourages each other to organize PA | Support and coordination among staff |
|  | LTCF residents are stimulated by seeing others performance | Involving residents and families |
|  | To reduce the care burden of the staff | Conviction that the guideline is evidence-based and will demonstrate improvement |
|  | Interaction with the family and the LTCF resident | Involving residents and families |
|  | The family of the LTCF resident asks to organize PA | Involving residents and families |
|  | Having appropriate material | Adequate services, resources, and time |
|  | PA is embedded in the vision of the LTCF / Part of the services that LTCF have to offer | Leadership and support/champion  Well-designed strategies, protocols, and resources |
|  | Organizing PA improves the image of the LTCF | Noticeable outcomes from guideline implementation |
|  | Having enough financial resources for organizing PA | Adequate services, resources, and time |
|  | Appropriate infrastructure in and outside the LTCF | Adequate services, resources, and time |
|  | Contact with the world outside the LTCF | Good communication and information flow |
| Kaasalainen et al. (2014) | Support from administration | Leadership and support/champion |
|  | Education for staff about pain management | Adequate education/training |
|  | Ability of nurse practitioners to order certain pain medications | Adequate services, resources, and time |
|  | Dedicated nurse specialist/practitioner for commitment of protocol (change champion) | Leadership and support/champion |
| Vikstrom (2015) | Experiencing everyday outcomes from the intervention (Staff related that interventions were often experienced positively, enriching everyday life) / if interpret: visible positive outcomes from interventions | Noticeable outcomes from guideline implementation |
| Lim et al. (2014) | Having fewer general practitioners with greater patient loads working in each facility would promote more consistent prescribing practices | Adequate services, resources, and time |
|  | Past experiences with quality improvement initiatives | Leadership and support/champion |
|  | Having nursing staff disseminate information and facilitation by onsite state | Support and coordination among staff |
| Dellefield et al. (2014) | Belief that pressure ulcers are preventable | Conviction that the guideline is evidence-based and will demonstrate improvement |
|  | Teamwork - e.g. relying on NAs to do early detection | Support and coordination among staff |
|  | Involvement of residents and families | Involving residents and families |
|  | Preventative care | Well-designed strategies, protocols, and resources |
|  | Communication between wound care team and staff | Good communication and information flow |
|  | Diverse educational strategies | Adequate education/training |
|  | Right amount and type of equipment | Adequate services, resources, and time |
| Berta et al. (2013) | Management ensures adequate resources available for implementing new/changed protocols | Adequate services, resources, and time  Leadership and support/champion |
|  | Management communicates reasons to staff for introducing new protocols/changing existing protocols | Good communication and information flow |
|  | Staff are provided time to attend in-services and to practice new knowledge | Adequate services, resources, and time |
|  | Staff are given an opportunity to discuss new protocols with management and to provide input into changes made to care practices and associated practice tools | Support and coordination among staff |
|  | Staff can clearly “see” a connection between the new protocol and improved resident outcomes | Noticeable outcomes from guideline implementation |
|  | Implementing new/changed protocols is seen to result in some real benefit to the staff themselves | Noticeable outcomes from guideline implementation |
|  | The literacy levels of staff are taken into account when developing implementation aids | Well-designed strategies, protocols, and resources |
|  | The experience levels of staff are taken into account when developing implementation aids | Well-designed strategies, protocols, and resources |
|  | Collaboration and sharing of experiences with new protocol is encouraged among staff/units | Support and coordination among staff  Good communication and information flow |
|  | A written implementation plan is developed to guide the protocol implementation | Well-designed strategies, protocols, and resources |
|  | A staff member is identified as a “champion” to guide the protocol implementation process | Leadership and support/champion |
|  | Data are collected on the impact of protocol and reported regularly to staff | Well-designed strategies, protocols, and resources |
|  | “Champions” are given adequate protected time and other resources to implement care protocols | Leadership and support/champion  Adequate services, resources, and time |
|  | Care protocols are assessed for their compatibility or similarity to others already in place | Well-designed strategies, protocols, and resources |
|  | “Champions” have prior experience and success with care protocol | Leadership and support/champion |
|  | Champions” are accessible to staff 24/7 | Leadership and support/champion  Adequate services, resources, and time |
| Kaasalainen et al. (2012) | Using APNs as onsite change champions | Leadership and support/champion |
|  | Management and senior leadership buy-in and support | Leadership and support/champion |
|  | Education about pain management | Adequate education/training |
|  | Staff value the topic and want to ensure residents' pain in well managed | Positive emotional responses to work and the intervention |
| Verkaik et al. (2011) | presence of an opinion leader | Leadership and support/champion |
|  | having a reporting system that guidelines fit into | Well-designed strategies, protocols, and resources |
|  | warm and creative personality | Positive emotional responses to work and the intervention |
|  | sharing interests with residents | Involving residents and families |
|  | visible reduction in depression | Noticeable outcomes from guideline implementation |
|  | positive attitudes of relatives | Positive emotional responses to work and the intervention |
| Berta et al. (2010) | Having in place a learning culture | Positive emotional responses to work and the intervention |
|  | Membership to a multi-unit/chain organization that afforded dedicated or shared resources for practicing change initiatives | Adequate services, resources, and time  Leadership and support/champion |
| McConigley et al. (2008) | parts of the guideline already being implemented | Well-designed strategies, protocols, and resources |
|  | knowledgeable about pain management techniques | Adequate education/training |
|  | resident assessment | Good communication and information flow |
|  | existing palliative care knowledge | Adequate education/training |
| Hilton et al. (2016) | Adequate equipment/products | Adequate services, resources, and time |
|  | family participation/support | Involving residents and families |
|  | family willing to pay for services | Adequate services, resources, and time |
|  | provide products/  equipment | Adequate services, resources, and time |
|  | Resident compliance | Involving residents and families |
|  | resident being able to participate in their own oral care (self-care abilities); | Involving residents and families |
|  | educating residents on their rights | Involving residents and families |
|  | Increased staff | Adequate services, resources, and time |
|  | increased time | Adequate services, resources, and time |
|  | specific staff designated responsibility of oral care | Support and coordination among staff |
|  | staff education/training | Adequate education/training |
|  | Improved organisational systems | Well-designed strategies, protocols, and resources |
|  | Written documentation/orders for each  resident | Well-designed strategies, protocols, and resources |
|  | Policy implementation/set oral care program as part of routine | Leadership and support/champion  Well-designed strategies, protocols, and resources |
| Lau et al. (2007) | Balance treatment guidelines with geriatric training and clinical experience when determining what medications would be appropriate for their residents. | Adequate education/training |
| Buss et al. (2004) | Familiarity with guidelines | Adequate education/training |
| Kong et al. (2020) | Care workers should have received education about person-centered dementia care | Adequate education/training |
|  | Need for family education about dementia and person-centred care | Adequate education/training |
|  | A need for support to improve their negative mindset | Support and coordination among staff |
|  | The importance of good relationship and cooperation between staff and family to provide person-centred care for residents with dementia | Support and coordination among staff |
| Eldh et al. (2020) | Resident-centredness | Involving residents and families |
|  | Physical environment | Innovative environmental modifications |
|  | Knowledge | Adequate knowledge and education |
| Cossette et al. (2019) | Commitment to the improvement | Positive emotional responses to work and the intervention |
|  | Involvement | Support and coordination among staff |
|  | Skills and capabilities of those involved | Adequate knowledge an education |
|  | Leadership | Leadership and champions |
|  | Team functioning | Support and coordination among staff |
|  | Resources in place | Adequate services, resources, and time |
|  | Progress monitored for feedback and learning | Noticeable outcomes from guideline implementation |
|  | Evidence of the benefits | Noticeable outcomes from guideline implementation |
|  | Robust and adaptable processes | Well designed strategies, protocols, and resources |
|  | Alignment with organizational culture and priorities | Well designed strategies, protocols, and resources |
|  | Support for improvement | Support and coordination among staff |
|  | Alignment with external political and financial environment | HC system structure – barriers? |
| Surr et al. (2020) | Leaders who valued the intervention | Leadership and champions |
|  | Recognizing the need for change | Positive emotional responses to work and the intervention |
|  | Selecting well-established staff as champions | Leadership and champions |
|  | Changing staff’s perception of the condition | Positive emotional responses to work and the intervention |
|  | Shared understanding of the need for improvements | Support and coordination among staff |
|  | Embedded DCM data, feedback, and action plans into the work of the care homes | Well-designed strategies, protocols, and resources |
| Desveaux et al. (2019) | Tailored home-level approaches | Well-designed strategies, protocols, and resources |
|  | Active one-on-one education | Adequate knowledge and education |
|  | Extending education to residents and family | Involving residents and families |
| Walker et al. (2019) | Improved knowledge | Adequate knowledge and education |
|  | Good relationship with and support from GPs and pharmacists | Support and coordination among staff |
|  | Support from family members | Involving residents and families |
|  | Peer pressure from other residents to consider vitamin D | Involving residents and families |
|  | Ongoing internal audits to identify residents that are not prescribed an adequate dose of vitamin D | Well-designed strategies, protocols, and resources |
